# Structure‐Activity Relationships in Nucleic‐Acid‐Templated Vectors Based on Peptidic Dynamic Covalent Polymers

**DOI:** 10.1002/chem.202202921

**Published:** 2022-12-12

**Authors:** Dan‐Dan Su, Lamiaa M. A. Ali, Maëva Coste, Nabila Laroui, Yannick Bessin, Mihail Barboiu, Nadir Bettache, Sébastien Ulrich

**Affiliations:** ^1^ IBMM Institut des Biomolécules Max Mousseron CNRS Université de Montpellier, ENSCM 34095 Montpellier France; ^2^ Institut Européen des Membranes Adaptive Supramolecular Nanosystems Group Université de Montpellier, ENSCM, CNRS Place Eugène Bataillon, CC 047 34095 Montpellier France; ^3^ Department of Biochemistry Medical Research Institute University of Alexandria 21561 Alexandria Egypt

**Keywords:** dynamic covalent polymers, nucleic acid complexation, peptides, siRNA delivery, templated self-assembly

## Abstract

The use of nucleic acids as templates, which can trigger the self‐assembly of their own vectors represent an emerging, simple and versatile, approach toward the self‐fabrication of tailored nucleic acids delivery vectors. However, the structure‐activity relationships governing this complex templated self‐assembly process that accompanies the complexation of nucleic acids remains poorly understood. Herein, the class of arginine‐rich dynamic covalent polymers (DCPs) composed of different monomers varying the number and position of arginines were studied. The combinations that lead to nucleic acid complexation, in saline buffer, using different templates, from short siRNA to long DNA, are described. Finally, a successful peptidic DCP featuring six‐arginine repeating unit that promote the safe and effective delivery of siRNA in live cancer cells was identified.

## Graphical abstract

Structure–activity Relationships in Nucleic‐Acid‐Templated Vectors Based on Peptidic Dynamic Covalent Polymers (M. Barboiu, N. Bettache, S. Ulrich et al.), @DDSU0407; @M_Cste; @MihailBarboiu; @IBMM_Balard

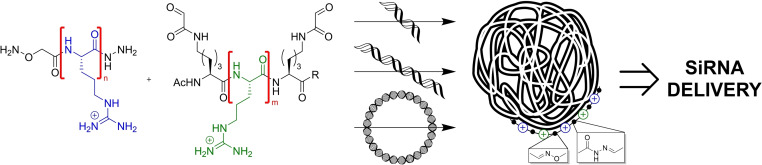

## Introduction

DNA condensation is a natural phenomenon used to store genetic information in compact nucleosomes. Thanks to the self‐assembly of histones into octamers, the whole three billion base pair human genome can be stored in a book fitting each of the 10 μm diameter cell nuclei.^[1]^ The dynamic control of gene expression is operated by the reversible supramolecular association of protein and enzyme complexes, and by the enzyme‐mediated dynamic covalent modifications of nucleic acids (DNA methylation) and proteins (histone acetylation and methylation).^[2]^ This dynamic modulation of DNA condensation enables the controlled expression of genetic information, whenever and wherever required, by the reversible switching between euchromatin (decondensed/accessible DNA, transcription possible) and heterochromatin (condensed DNA, transcription prevented) structures. These examples of responsive DNA complexation by reversible supramolecular assemblies, along with the other examples of viruses as dynamic nucleic acid packaging and delivery devices,^[3]^ are a source of inspiration for the fabrication of artificial gene delivery vectors displaying dynamic non‐covalent or reversible covalent linkages.

In this context, self‐assembly processes are also promising for generating, in a simple and versatile manner, smart vectors of therapeutic nucleic acids (e.g. DNA; short‐interfering RNA, siRNA; messenger RNA, mRNA)^[4]^ that adapt their constitution during 1) nucleic acid complexation (template effect), and 2) nucleic acid release which may be responsive to physico‐chemical cues.^[5]^ Hitherto, only a few examples of such dynamic vectors – undergoing adaptation to nucleic acid complexation and capable of delivering siRNA in live cells – have been reported: amphiphilic dendrimers^[6]^ and self‐complementary cyclodextrins,^[7]^ redox‐sensitive poly‐disulfides,^[8]^ pH‐sensitive dynamic conjugates^[9]^ and dynamic covalent polymers.^[10]^

However, the templating effects of nucleic acids can follow complex rules. While the local structure and persistence length of double‐stranded DNA are well understood and can be used to predict the binding of small molecules,^[11]^ the template effect leading to the formation of a complexing agent that condenses nucleic acids is more delicate to apprehend. In fact, the binding of polycations induces a charge compensation that reduces the electrostatic repulsion between negatively‐charged phosphate groups in nucleic acids, hence decreases the persistence length and triggers condensation.^[12]^ Template effects can therefore occur on the initially extended DNA but also on partially condensed states along the DNA compaction pathway following different rules.^[13]^

Multivalency plays a key role in DNA condensation since 90 % of its charge must be neutralized by counterions to provoke condensation, meaning that cationic species of valency lower than three cannot trigger DNA condensation in physiological saline conditions.[^13^, ^14^] For instance, trivalent cations such as cobalt(III) hexaamine or spermidine effectively condense DNA while divalent inorganic cations such as Mg^2+^ cannot. Another factor that can affect the nucleic acid template effect is the inter‐phosphate spacing that is shorter in RNA (ca. 5.5 Å) than in DNA (ca. 6.5 Å) due to the different sugar puckers – respectively C3’‐endo and C2’‐endo.^[15]^ The situation becomes even more complex for cationic ligands since their covalent scaffold has to position the cationic charges at appropriate positions that match the inter‐phosphate spacing of DNA in its condensed state. Finally, it is important to note that the template effect may originate from template‐accelerated kinetics of assembly between monomers. It directly follows that there should be a threshold for the residence time of DNA‐bound monomers, and hence a threshold value for the binding constants of monomers onto DNA that permit this template effect to occur.

To our knowledge, there are only three examples of cationic dynamic covalent polymers which formation was shown to be templated by nucleic acids: in 2015 the group of Aida used oxidative polymerization of dithiol monomers bearing three or four guanidinium groups spaced by a 7.4 Å flexible spacer,^[8c]^ followed in 2019 by the group of Li and Yang who used ring‐opening disulfide‐exchange polymerization of a guanidinium disulfide monomer,^[8b]^ and in 2021 our group reported siRNA‐templated polymerization of glycosylated peptide‐based dynamic covalent polymers, made of complementary arginine‐rich modified peptides, for the cell‐selective delivery of siRNA.^[10a]^ Despite these successful proof‐of‐concepts that follow different designs, a systematic study of structure‐activity relationships is unreported. Herein, we investigated the nucleic acid‐templated formation of arginine‐rich dynamic covalent polymers. We studied different monomers varying the number and position of arginines and provide structure–activity relationships for DNA as well as siRNA complexation. Finally, the selected hits were used to successfully perform siRNA delivery in live cancer cells.

## Results and Discussion

### Design and synthesis

**Figure 1:**
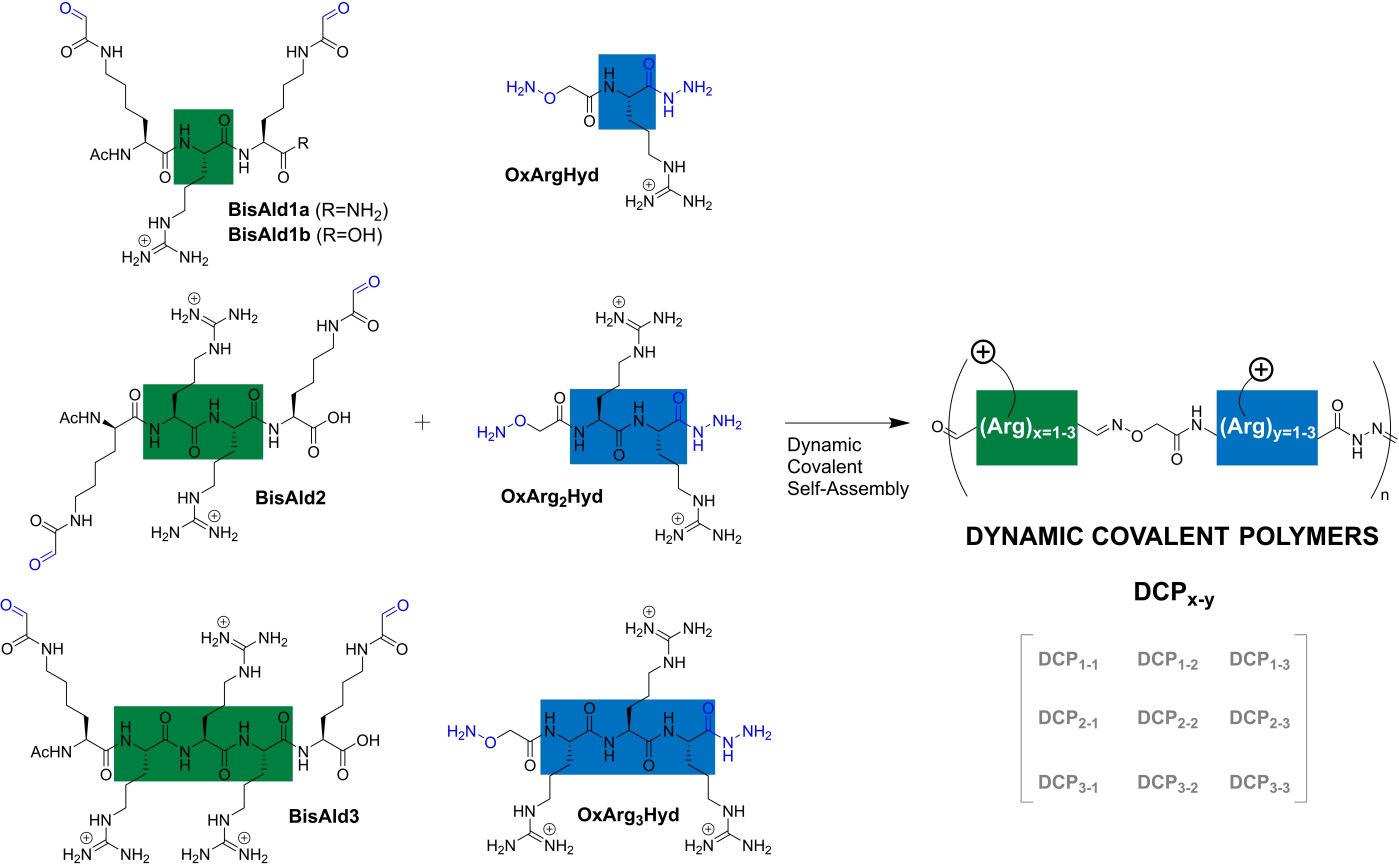
Structures of peptide building blocks and dynamic covalent polymers formed thereof. The colored blocks highlight the varying number of arginine residues. Ac: acetyl.

Our design of cationic dynamic covalent polymers (DCPs) takes inspiration from peptides known to interact with nucleic acids.^[16]^ It combines arginine‐rich peptide bisaldehydes **BisAld_n_** with complementary N‐aminooxy, C‐hydrazide arginine‐rich peptides **OxArg_n_Hyd**, resulting in alternate DCPs with precise equimolar amounts of the two building blocks (Figure 1). Since the multivalent presentation of cationic arginines is of prime importance for both nucleic acid complexation and cell penetration, we screened here nine different peptide monomers by varying the number of arginine between one and three, thus yielding cationic DCPs with repeating units made of two up to six arginines.

**Scheme 1:**
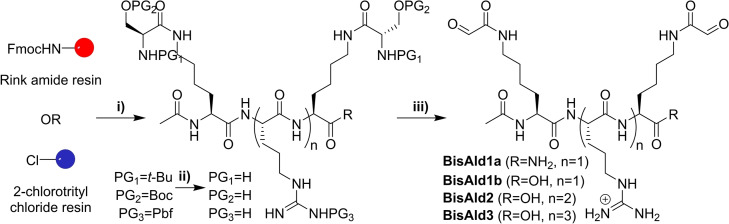
General synthetic scheme for the preparation of bisaldehyde peptides. i) SPPS, ii) deprotection in TFA/TIS/H_2_O 95/2.5/2.5; iii) oxidative cleavage with NaIO_4_. Amide C‐termini (R=NH_2_) obtained when starting from the Rink amide resin, carboxylic acid C‐termini (R=OH) obtained when starting from the 2‐chlorotrityl chloride resin. Boc: *tert*‐butyloxycarbonyl; Pbf: 2,2,4,6,7‐pentamethyIdlhydrobenzofuran‐5‐sulfonyl.

The bisaldehyde peptides were initially synthesized by solid phase peptide synthesis (SPPS) starting from a Rink amide resin, which yields C‐amide termini after one‐pot cleavage and deprotection in TFA/TIS/H_2_O 59/2.5/2.5 (Schemes 1, S1 and Figures S1–S18). Although we succeeded in preparing **BisAld1a** using this approach, it failed for making the highly polar **BisAld2** and **BisAld3** compounds due to reverse‐phase HPLC purification issues. Therefore, we resorted to using an acid‐sensitive 2‐chlorotrityl chloride resin which allowed the cleavage under mild conditions (TFA/CH_2_Cl_2_ 1/99 for 5 minutes) without deprotecting the peptides, thereby facilitating the purification and isolation of the protected peptides by preparative reverse‐phase HPLC, before carrying the deprotection step using TFA/TIS/H_2_O 95/2.5/2.5. Finally, the oxidative cleavage of the serine side‐chains was performed using sodium periodate and afforded the desired bisaldehyde peptides.

**Scheme 2:**
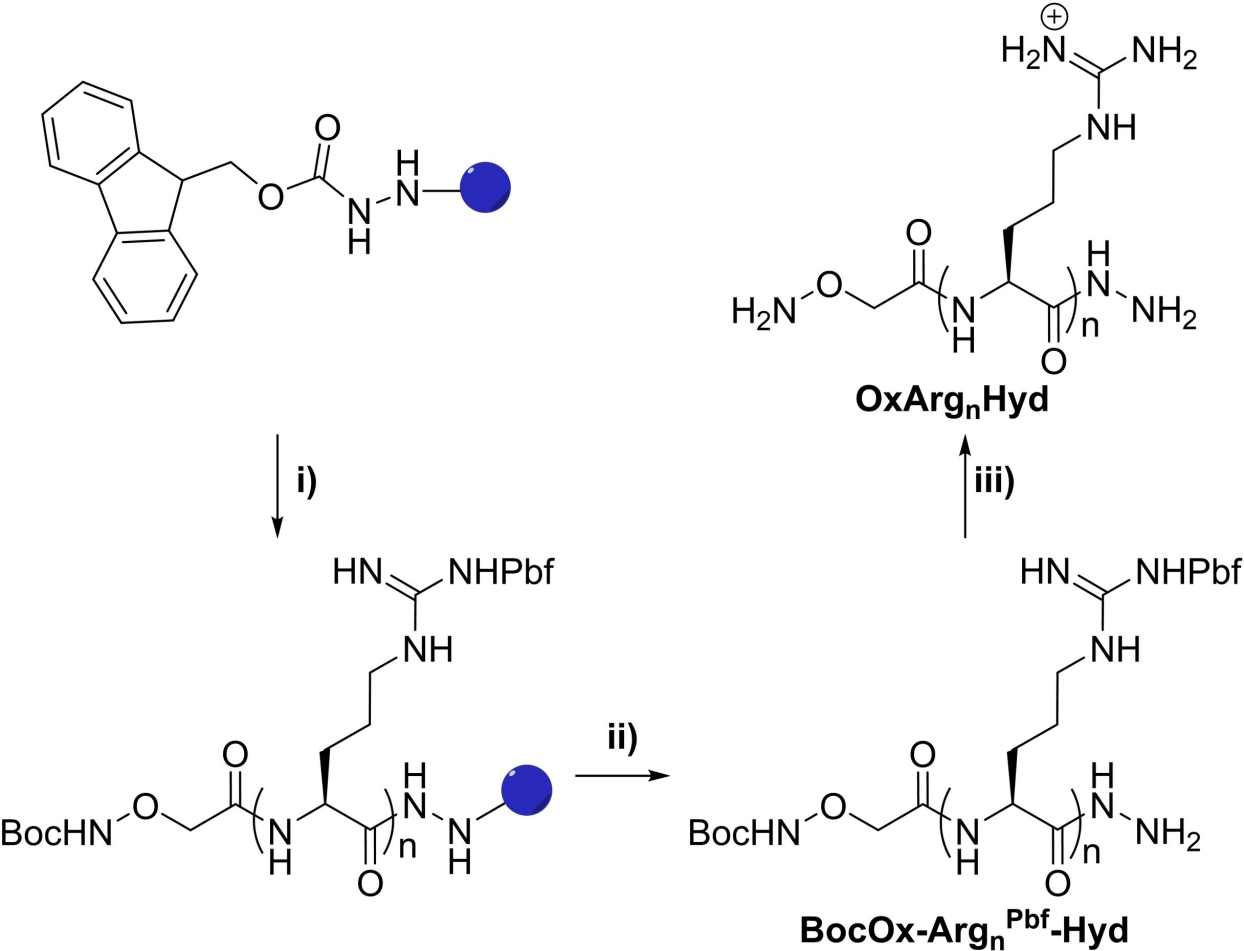
General synthetic scheme for the preparation of **OxArg_n_Hyd**: i) solid‐phase peptide synthesis, ii) mild cleavage, iii) deprotection. Pbf: 2,2,4,6,7‐pentamethyIdlhydrobenzofuran‐5‐sulfonyl; Boc: *tert*‐butoxycarbonyl.

The N‐aminooxy, C‐hydrazide peptides **OxArg_n_Hyd** were prepared using a solid‐phase peptide synthesis approach that was derived from our previous solution phase approach (Scheme 2 and Figures S19–S27).^[10b]^ In short, a 2‐chlorotrityl chloride resin was modified with a Fmoc carbazate,^[9c]^ then the peptide sequence was built, and finally a coupling with the N‐hydroxysuccinimide activated ester of N‐Boc aminooxyacetic acid was carried out. A mild cleavage afforded the desired protected peptides which were isolated by reverse‐phase HPLC, before carrying out the final deprotection to yield the final **OxArg_n_Hyd** after precipitation in diethylether.

### Screening of DCPs for DNA‐templated complexation

**Figure 2:**
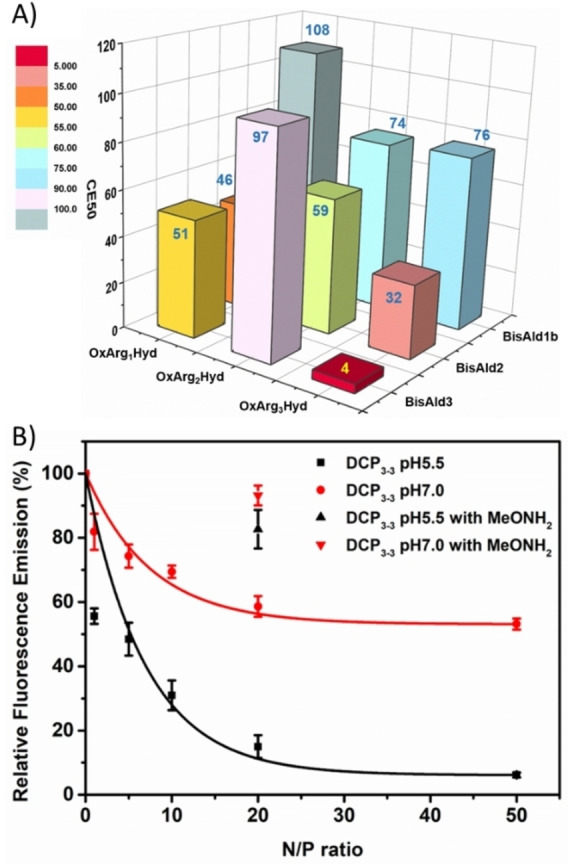
Complexation of ctDNA by **DCP_x‐y_** analyzed by fluorescence displacement assay: A) CE_50_ of **DCP_x‐y_** formed through in situ templated polymerization of monomers **BisAldn** and **Ox‐Arg_n_‐Hyd** in saline sodium acetate buffer (100 mM AcONa, 10 μM EDTA, 150 mM NaCl, pH 5.5); B) Relative fluorescence emission of ethidium bromide as a function of the N/P ratio for the ctDNA complexation by **DCP_3‐3_** at two different pH (5.5 vs. 7.0); the relative fluorescence emissions are also represented for the same experiments carried out at N/P=20 in the presence of 100 equiv. methoxyamine MeONH_2_.

DNA complexation through in situ templated polymerization was first assessed by a fluorescence displacement assay using ethidium bromide as a fluorophore that lights up upon intercalation into calf thymus DNA (ctDNA).^[17]^ Complexation is here detected by a decrease in fluorescence emission of the probe. Varying amounts of the two monomers **BisAldn** and **Ox‐Arg_n_‐Hyd** in a 1 : 1 stoichiometric ratio were incubated in saline sodium acetate buffer (100 mM AcONa, 10 μM EDTA, 150 mM NaCl, pH 5.5) in the presence of ctDNA for 16 h. The results are given as Charge Excess 50 (CE_50_) which corresponds to the nominal number of positive charges excess brought by the DCPs, relative to the number of negative charges present in ctDNA, which is required to trigger a 50 % decrease in the fluorescence intensity. The results presented in Figure 2A clearly reveal a strong effect of the number of arginines, **DCP_1‐1_** being the least active (CE_50_=108), while **DCP_3‐3_** is the most active (CE_50_=4) (see also Figure S28). There is a general trend of improving ctDNA complexation when increasing the number of arginines in either of the building blocks. For comparison, the CE_50_ of spermine – a well‐known biogenic ligand of dsDNA involved in cell growth^[18]^ – is around 1500 in saline (150 mM NaCl) buffer.[^10c^, ^19^]

Looking more specifically at the best hit, **DCP_3‐3_**, we found the CE_50_ to be pH‐dependent and much weaker when the in situ templated polymerization was carried out at neutral pH (Figure 2B). Since the pH is not expected to significantly alter the degree of protonation of arginine‐rich peptides due to the high pKa (ca. 13) of the guanidinium group, this behavior is interpreted by the polycondensation being slower at neutral pH compared to acidic pH – a well‐established fact for acylhydrazone and oxime conjugation reactions.^[20]^ Furthermore, when the same experiment at N/P=20 (N/P ratio corresponds to the molar ratio of positively‐charged guanidiniums brought by the peptide monomers per phosphodiester group in the nucleic acid) was carried out in the presence of an excess of methoxyamine (100 equiv.), acting as a terminator of the polymerization process, a strong increase in the relative fluorescence emission was observed: 82.7±6.0 % and 93.2±3.1 % at pH 5.5 and 7.0, respectively, after 16 h incubation (Figure 2B). This confirms the central role of the polycondensation in the observed DNA complexation.

**Figure 3:**
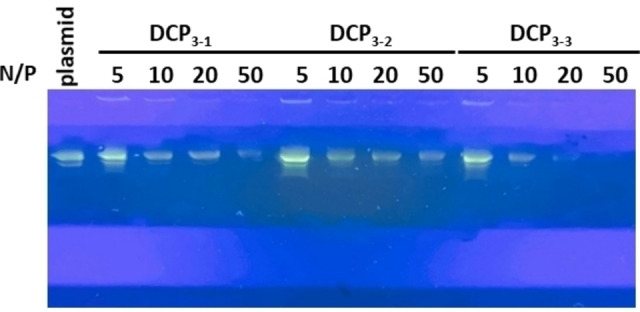
Gel electrophoresis of pDNA with **DCP_x‐y_** formed through in situ templated polymerization of monomers **BisAldn** and **Ox‐Arg_n_‐Hyd** at different N/P.

Those results were cross‐checked using gel retardation assays carried out with plasmid DNA (pDNA), a circular double‐stranded DNA most suited for gel electrophoresis, screening different N/P ratios where polyplexes were previously formed and incubated for 16 h in saline sodium acetate buffer (100 mM AcONa, 10 μM EDTA, 150 mM NaCl, pH 5.5). The results confirmed that **DCP_3‐3_** is the most active, reaching full complexation at N/P=20 (Figures 3 and S29), while both building blocks **OxArg_3_Hyd** and **BisAld3** remain ineffective up to N/P=100 (Figure S30). The use of **DCP_3‐3_** pre‐formed at high concentration (50 mM) prior to mixing with pDNA gave a similar result, thereby confirming that complexation occurs in situ as a result of templated DCP formation (Figure S31).

Next, we characterized the polyplex formed between **DCP_3‐3_** and a short (21 bp) double‐stranded DNA (dsDNA) of the same length and sequence as an siRNA of interest (see below) by dynamic light scattering (DLS), ζ potential measurements, and Transmission Electron Microscopy (TEM). The DLS results showed nanoparticles size in the range 250–590 nm depending on the mode of preparation (Table 1). While using pre‐formed **DCP_3‐3_** or a pre‐equilibrated library of **BisAld3** and **Ox‐Arg_3_‐Hyd** yields smaller and similar particles, the in situ templated polymerization produced the larger species. ζ potential measurements confirmed DNA complexation and a tendency to charge neutralization as previously observed for this templated polymerization driven by electrostatic binding (Table 1).^[10a]^ Only using pre‐formed **DCP_3‐3_** gave a positive ζ potential, revealing that its positive charge number slightly exceeds the negative charge numbers of dsDNA.

**Table 1:** Characterization of particle size, polydispersity index (PDI) and ζ‐potential for dsDNA polyplexes induced by **DCP_3‐3_** at N/P=20.

Sample/conditions	Size [nm]	PDI	Zeta‐potential [mV]
dsDNA	473±74	0.58±0.20	−3.3±0.9
**DCP_3‐3_**	397±114	0.43±0.09	0.5±0.3
*In situ* templated polymerization^[a]^	588±78	0.69±0.33	−1.8±0.6
Pre‐equilibrated library^[b]^	262±50	0.32±0.16	−1.3±1.3
Pre‐formed **DCP_3‐3_** ^[c]^	248±53	0.26±0.07	1.8±0.8

[a] *In situ* templated polymerization of **DCP_3‐3_** by dsDNA; ^b^ **BisAld3** and **Ox‐Arg_3_‐Hyd** pre‐equilibrated before the addition of dsDNA; ^c^ **DCP_3‐3_** formed at high concentration (50 mM) prior to adding dsDNA. [b] **BisAld3** and **Ox‐Arg_3_‐Hyd** pre‐equilibrated before the addition of dsDNA. [c] **DCP_3‐3_** formed at high concentration (50 mM) prior to adding dsDNA.

**Figure 4:**
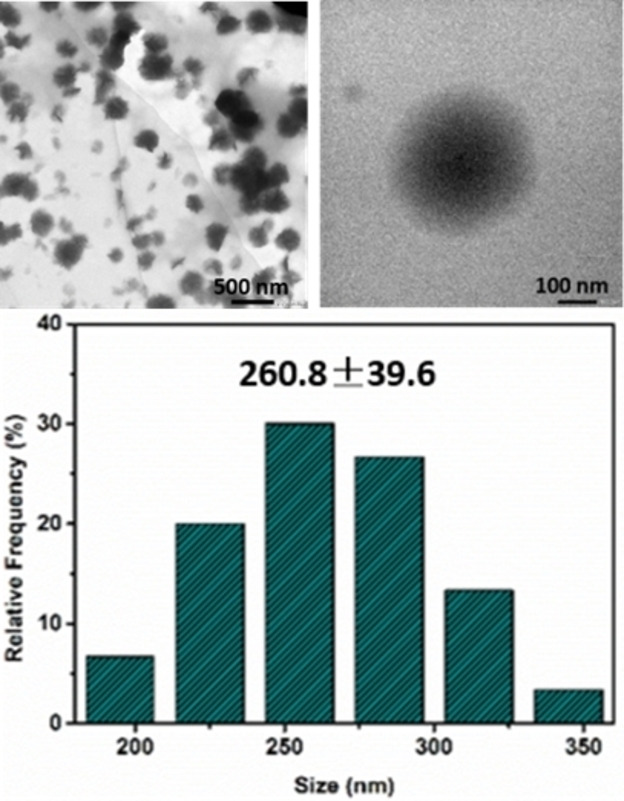
Representative TEM micrographs (top) and size analysis (bottom) for dsDNA polyplexes induced by **DCP_3‐3_** at N/P=20.

TEM analyses showed spherical nanoparticles for the polyplexes prepared by in situ templated polymerization, with apparent sizes centered around 250–300 nm (Figure 4).

### Screening DCPs for siRNA complexation

**Figure 5:**
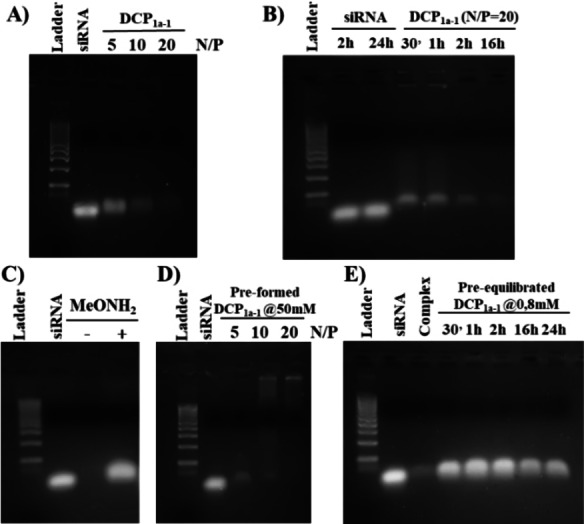
Gel electrophoresis showing siRNA complexation by **DCP_1a‐1_**: A) at different N/P ratio following 16 h incubation; B) time‐course at N/P=20; C) at N/P=20, and 16 h incubation in absence (−) and presence (+) of methoxyamine; D) using **DCP_1a‐1_** preformed at high concentration (50 mM); E) from the library of **BisAld1a** and **Ox‐Arg‐Hyd** pre‐equilibrated at low concentration.

We initially started our study of siRNA complexation with the simplest and most affordable building blocks: **BisAld1a** and **Ox‐Arg‐Hyd**. After 16 h of incubation with siRNA, gel electrophoresis showed complete complexation at N/P=20, with complexation occurring gradually over this time course (Figures 5A and 5B). SiRNA complexation was found to be complete starting with **DCP_1a‐1_** preformed at high concentration (50 mM), and did not to occur from the library of **BisAld1a** and **Ox‐Arg‐Hyd** pre‐equilibrated at low concentration, or in the presence of the polymerization terminator methoxyamine (Figures 5C–E). All these results confirm that **DCP_1a‐1_** forms in a siRNA‐templated polymerization process.

**Figure 6:**
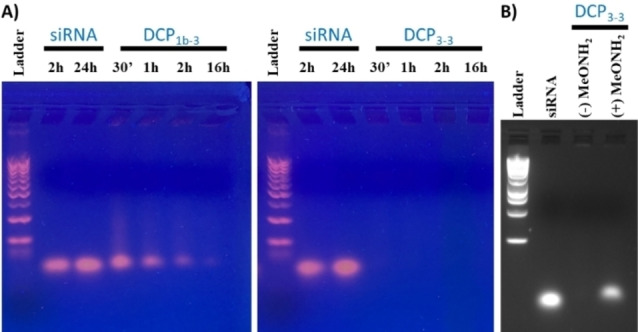
A) Monitoring of the siRNA complexation through in situ templated polymerization process by **DCP_1b‐3_** (left) and **DCP_3‐3_** (right) at N/P=20 at different incubation times in saline sodium acetate buffer (100 mM AcONa, 10 μM EDTA, 150 mM NaCl, pH 5.5). B) Role of the polycondensation on siRNA complexation by **DCP_3‐3_** (N/P=20, 30 minutes incubation) shown by comparing reactions carried out in absence (‐) and presence (+) of methoxyamine.

Investigating building blocks featuring multiple arginines showed a complete complexation of siRNA at N/P>20 by **DCP_3‐3_** (Figure S32 and S33). Interestingly, we found that increasing the numbers of arginines in one or both building block(s) significantly accelerates the in situ templated polymerization process – complete siRNA complexation occurring after only 30 minutes incubation using **DCP_3‐3_** (Figure 6A), compared to the 16 h required for **DCP_1a‐1_** or **DCP_1b‐3_**. However, even in the case of **DCP_3‐3_**, a complexation promoted by the non‐covalent assembly of individual peptides onto siRNA is rule out since the experiment using methoxyamine (167 equiv.) as a polycondensation terminator confirms the prime role of the covalent assembly of the peptides on the observed siRNA complexation (Figure 6B).

The siRNA polyplexes were analyzed by DLS and ζ potential measurements (Table 2). Although the polyplexes formed using **DCP_1b‐3_** gave polydisperse nanoparticles which precise characterization was not even possible, the polyplexes formed using **DCP_3‐3_** gave nanoparticles of similar size and ζ potential compared to those previously obtained with dsDNA (see Table 1). Also, the polyplexes formed using **DCP_1a‐1_** have similar size and ζ potential which is remarkable considering that the monomers bear a different number of cationic arginine residues, suggesting that the degree of polymerization adapts until charge compensation is reached.

**Table 2:** Characterization of particle size, polydispersity index (PDI) and ζ‐potential for siRNA polyplexes induced by **DCP_x‐y_** at N/P=20.

Sample/conditions	Size [nm]	PDI	Zeta‐potential [mV]
**DCP_1a‐1_**	309±239	0.45±0.12	3.0±4.3
**DCP_1b‐3_**	n.d.^[a]^		
**DCP_3‐3_**	341±94	0.37±0.10	−2.3±0.6

[a] n.d.: not detected, sample too polydisperse.

### SiRNA delivery in live cells

**Figure 7:**
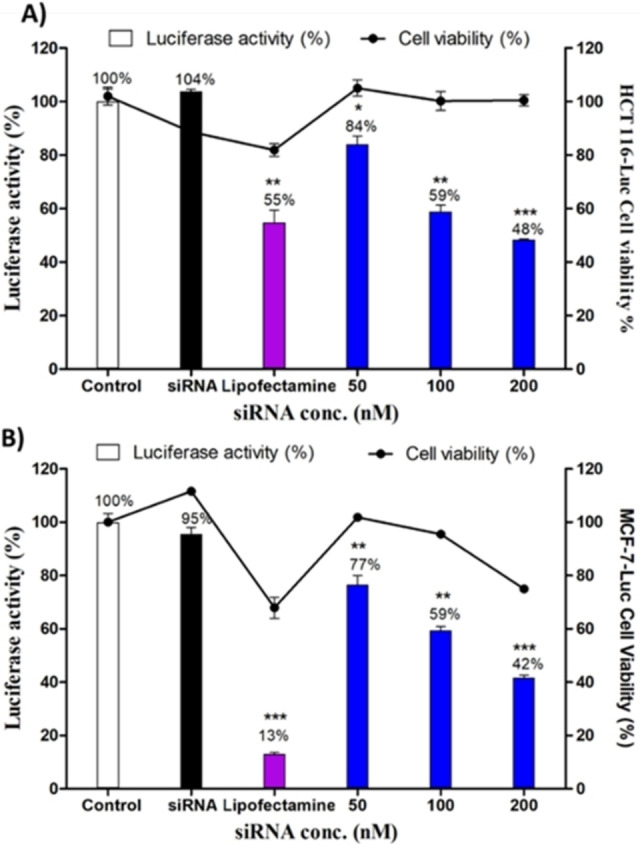
Knock‐down of luciferase activity and cell viability using **DCP_3‐3_** on A) HCT116‐Luc cells, and B) MCF‐7‐Luc cells after 72 h of incubation with siRNA‐**DCP_3‐3_** polyplex. The DCPs were made in situ from siRNA‐templated polymerization as previously reported.^[10a]^ White bar: cells without any treatment; black bar: cells incubated with siLuc alone at 100 nM; purple bar: lipofectamine; then **DCP_3‐3_** (N/P=20) with siLuc concentrations of 50, 100, and 200 nM (blue bars). The luciferase activity was adjusted depending on cell viability of each condition. The results were the average of three independent experiments and presented as mean ± SEM. * statistically significant from control (p<0.05), ** p<0.005 and** p<0.0005.

We first tested single arginine‐containing building blocks for siRNA‐Luc delivery in live cells, which was quantified by the knock‐down of luciferase activity. However, in this series, no activity was detected on colorectal (HCT116‐Luc) or breast (MCF‐7‐Luc) cancer cell lines (Figure S34). Similarly, introducing three arginines on one peptide building block was not sufficient as no activity was observed when testing **DCP_1b‐3_** on HCT116‐Luc cells (Figure S35). The introduction of three arginines on the second peptide building block led to a detectable dose‐dependent activity on both HCT116‐Luc and MCF‐7‐Luc cells, the luciferase activity decreased to 48±0.2 % and 42±1 %, respectively at 200 nM siRNA concentration (Figure 7). **DCP_3‐3_** acts therefore as an effective vector of siRNA, which is less cytotoxic than lipofectamine – cell viability remaining, at the maximum concentration tested (28 μM), at 100 % and 75±1% in the two different cell lines, respectively, HCT‐116‐Luc and MCF‐7‐Luc. Since **DCP_1a‐1_** and **DCP_3‐3_** form nanoparticles of similar size and ζ potential, we interpret the difference of efficiency in siRNA delivery on the basis of multivalent binding and cell penetration. We propose that **DCP_3‐3_** has monomers interacting through multiple salt bridge interactions with siRNA, thus allowing the flipping of some arginines for promoting cell internalization without compromising the nucleic acid binding. On the other hand, such flipping of arginines outside of the polyplex formed by the weaker binder **DCP_1a‐1_** may result in premature siRNA release and subsequent depolymerization of the vector, thus explaining its lower performance.

## Conclusion

We reported a synthetic methodology for making peptide bisaldehydes **BisAld_n_** and complementary N‐aminooxy, C‐hydrazide arginine‐rich peptides **OxArg_n_Hyd**, which both take part in the formation of peptide‐based dynamic covalent polymers (DCPs). The approach is versatile and will enable inserting different sequences in the future.^[21]^ For now, we have explored arginine‐rich DCPs that are formed in situ by nucleic acid‐templated polymerization. Compared to the robotic screening of polymers for transfection applications, which require cumbersome work,^[22]^ our methodology based on templated self‐assembly simply involves mixing of appropriate building blocks at room temperature in a slightly acidic saline buffer. Using long DNA (ctDNA, pDNA) as templates, only the DCP made of a six‐arginine repeating unit (**DCP_3‐3_**) stood out as an effective complexing agent. Using shorter siRNA as templates, complexation was evidenced with **DCP_1a‐1_** (two‐arginine repeating unit), **DCP_1b‐3_** (four‐arginine repeating unit), and **DCP_3‐3_** (six‐arginine repeating unit). However, only the latter was shown to be able to deliver siRNA effectively and safely in live cells, with an activity rivalling lipofectamine and a greater cytocompatibility. This work provides valuable information on the structure–activity relationships that govern the nucleic acid‐templated polymerization of arginine‐rich DCPs. Compared to our previous result proving the concept of siRNA‐templated dynamic covalent polymerization,^[10a]^ this work extends it to longer nucleic acids. The results described herein shall inspire future studies implementing DCPs for the delivery of nucleic acid therapeutics such as short siRNA or longer DNA or mRNA.^[4b]^

## Experimental Section

**General procedures and materials**: All reagents and solvents were obtained from commercial sources and were used without further purification. The dipeptide Fmoc‐*l*‐Lys[Boc‐*l*‐Ser(O^t^Bu)]OH^[23]^ and N’‐Boc‐aminooxyacetyl N‐hydroxysuccinimide ester^[24]^ were synthesized following previously reported protocols.^[9c]^ The Fmoc‐protected hydrazine resin (0.595 mmol/g) was prepared from the 2‐chlorotrityl chloride resin (loading; 1.60 mmol Cl/g) as previously described.[^9c^, ^25^] DsDNA sequences: sequence 1 (5’‐CGT‐ACG‐CGG‐AAT‐ACT‐TCG‐ATT‐3’); sequence 2 (3’‐TCG‐AAG‐TAT‐TCC‐GCG‐TAC‐GTT‐5’). The different siRNA sequences are for anti‐firefly luciferase (siLuc): 5′ CUU‐ACG‐CUG‐AGU‐ACU‐UCG‐AdTdT‐3’ (sense strand), and 5’‐UCG‐AAG‐UAC‐UCA‐GCG‐UAA‐GdTdT‐3’ (anti‐sense strand) and for siRNA without biological activity, used as control (siCtrl): 5’‐CGU‐ACG‐CGG‐AAU‐ACU‐UCG‐AdTdT‐3’ (sense strand) and 5’‐UCG‐AAG‐UAU‐UCC‐GCG‐UAC‐GdTdT‐3’ (anti‐sense strand) were purchased from Eurogentec (Serring, Belgium). Lipofectamine RNAiMAX was purchased from Invitrogen (Cergy Pontoise, France). d‐luciferin potassium salt was Purchased from PerkinElmer (Waltham, USA). Cell viability reagent 3‐(4,5‐dimethylthiazol‐2‐yl)‐2,5‐diphenyltetrazolium bromide (MTT) was purchased from Sigma‐Aldrich (Saint‐Quentin‐Fallavier, France).

**Nuclear magnetic resonance spectroscopy (NMR)**: ^1^H and ^13^C NMR spectra were recorded at 400 MHz for ^1^H and 100 MHz for ^13^C (Bruker Avance 400 or ARX instruments) in deuterated solvents. Peaks were referenced in ppm with respect to the residual solvent peak. Data are reported as follows: chemical shift (δ in ppm), multiplicity (s for singlet, d for doublet, t for triplet, m for multiplet), coupling constant (*J* in Hertz), and integration.

**High‐performance liquid chromatography (HPLC)**: Analytical reverse‐phase HPLC (RP‐HPLC) analyses were performed on a Waters HPLC 2695 (EC Nucleosil 300–5 C18, (125×3 mm) column, Macherey‐Nagel) equipped with a Waters 996 DAD detector with the following linear gradients of solvent B (acetonitrile 100 %) into solvent A (TFA 95 % and acetonitrile 5 %): Method: 0 to 95 % of solvent B in 5 min; flow: 1 mL/min. Retention times (t_R_) are given in minutes. Preparative HPLC was performed on i) a Waters Prep LC Controller HPLC (XSelect CSH Prep C18, 5 μm,(250×30 mm) column, Macherey‐Nagel) equipped with a Waters 2489 detector, flow 30 min/mL [HLPC 1], or on ii) a VWR International LaPrep pump P110, a VWR LaPrep P314 Dual l absorbance detector and EZChrom software (15 C_18_ reversed‐phase column Waters x‐bridge, RP‐18, 25×250 mm, 5 μm), flow 40 min/mL [HPLC 2], using a binary gradient elution. HPLC eluents: (solution A: 99.9 % water, 0.1 % TFA; solution B: 99.9 % acetonitrile, 0.1 % TFA).

**Liquid chromatography‐mass spectrometry (LC/MS)**: Analyses were performed on a Shimadzu LCMS2020 (Phenomex Kenetex C18, 2.6 μm×7.5 cm, 100 Å) equipped with a SPD−M20 A detector with the following linear gradient of solvent B (99.9 % acetonitrile, 0.1 % HCOOH) and solvent A (99.9 % water and 0.1 % HCOOH): 5 to 95 % of solvent B in 5 min; flow 1 mL/min. Retention times (t_R_) are given in minutes.

**Mass spectrometry (MS)**: Electrospray ionization (ESI‐MS) analyses were carried out at the Laboratoire de Mesures Physiques, IBMM, Université de Montpellier using Micromass Q‐Tof instruments.

**Ethidium bromide fluorescent displacement assay**: The complexation ability of DCPs toward calf thymus DNA (ctDNA) was evaluated by a fluorescence displacement assay. Stock solutions of **BisAldn** and **Ox‐Arg_n_‐Hyd** at 10 mM were prepared in either sodium acetate buffer (100 mM sodium acetate, 10 μM EDTA, 150 mM NaCl, pH 5.5) or HEPES buffer (100 mM HEPES, 10 μM EDTA, 150 mM NaCl, pH 7.0). Then, **BisAldn** and **Ox‐Arg_n_‐Hyd** (1 : 1 stoichiometry) were mixed in the corresponding buffer with calf‐thymus DNA (ctDNA, Sigma‐Aldrich, 13.3 μg/mL final concentration) at the corresponding N/P ratio in a 96‐well plate. 30 μL of a 50 μM ethidium bromide stock solution was added into the above mixture (5 μM final concentration) to obtain 300 μL of final volume. After 16 h incubation time, the fluorescence data were obtained using a SAFAS Xenius spectrofluorometer. Excitation wavelength was set as 480 nm and the emission was measured at 620 nm. The relative fluorescence emission was calculated as below:

$$Relative\;fluorescence\;emission=\;\frac{I_{2}-I_{0}}{I_{1}-I_{0}}$$

I_2_ represents the fluorescence emission of DCPs‐complexed DNA and ethidium bromide; I_1_ represents the fluorescence emission of DNA and ethidium bromide; I_0_ represents the fluorescence emission of ethidium bromide.

**Gel retardation assay for pDNA complexation**: The complexation ability of all samples with pET‐15b plasmid DNA (5708 bp) at pH 5.5 was tested at different N/P ratio. Final volume of 10 μL mixture of pDNA (100 ng) in sodium acetate buffer (100 mM sodium acetate, 10 μM EDTA, 150 mM NaCl, pH 5.5) was prepared. Then, both building blocks (**BisAld_n_** and **Ox‐Arg_n_‐Hyd**) were added in 1 : 1 stoichiometric ratio and the mixture was incubated for 16 h. Then 2 μL of Blue 6×loading dye (Fisher Scientific) was added and run on a 0.7 % (w/v) agarose gel (50 V) for 20 min. The blue‐stained pDNA was visualized with SYBER Safe (Life Technologies) and imaged by using the ECX‐F20.L UV transilluminator (Vilber Lourmat, France), then the gel images were obtained with a smartphone camera.

**Gel retardation assay for siRNA complexation**: In RNase free water, a fixed concentration, 2 μM, of siCtrl was mixed with the appropriate amounts of DCP in order to reach an N/P ratio of 5, 10 and 20 and incubated for different time intervals (30 min, 1 h, 2 h, 16 h and 24 h) in sodium acetate buffer (100 mM sodium acetate, 10 μM EDTA, 150 mM NaCl, pH 5.5) at 25 °C. After incubation, xylene cyanol (0.25 %) loading dye was added to the mixture. Electrophoresis was carried out on a 2 % (w/v) agarose gel mixed with GelRed^TM^ nucleic acid gel stain (Interchim, France) in 1X TBE buffer (90 mM Tris‐borate/2 mM EDTA, pH 8.2). The gel was run in 0.5X TBE at 50 V for 1 h. A 100 bp DNA ladder from Sigma‐Aldrich (Saint‐Quentin‐Fallavier, S4 France) was used as a reference for the gel. The GelRed‐stained siRNA was visualized using a TFX‐20M model‐UV transilluminator (Vilber Lourmat, Marne‐la‐Vallée, France) and gel photographs were obtained with a smartphone camera.

**Dynamic light scattering (DLS) and ζ‐potential measurements**: Measurements were performed using Zetasizer Nano‐ZS instrument (Malvern, United Kingdom) and DTS 1070 zeta potential cells transparent ZEN0040 disposable micro‐cuvette at 25 °C. For DLS measurements, the dsDNA complexes and siRNA (siCtrl) complexes at N/P=20 were prepared in sodium acetate buffer solution (100 mM sodium acetate, 10 μM EDTA, 150 mM NaCl, pH 5.5) and incubated 16 h at a final volume of 1 mL. For ζ‐potential analyses, complexes were prepared as previously described. Three measurements with 12 runs for each were performed, and the data report the average and the standard deviation.

**Transmission electron microscopy (TEM)**: TEM images were obtained by using a JEM 1400+ electron microscopy, at Microscopie Electronique et Analytique (MEA), Université de Montpellier. 20 μL of the sample was dropped on a carbon coated copper grid and dried at room temperature, then the samples were observed at a 120 kV acceleration voltage at 25 °C.

**Cell culture**: Two cancer cell lines were used in this study: human colorectal cancer (HCT 116‐Luc) cell line, which was obtained from IRCM Cell Culture Unit (Montpellier, France), and human breast cancer (MCF‐7‐Luc) cell line, which was generously provided by Dr. P. Balaguer (ICM Montpellier, France) and derived from MCF‐7 cells by stable transfection of firefly luciferase gene (PCDNA 3.1 CMV‐Luc‐SVNeo). HCT 116‐Luc cells were grown in McCoy′s 5 A (Modified) Medium supplemented with 10 % fetal calf serum (FCS) and 1 % gentamycin. MCF‐7‐Luc cells were grown in F12/Dulbecco's modified Eagle's medium (DMEM) supplemented with 10 % FCS and 1 % geneticin. All cell lines were incubated at 37 °C in a humidified atmosphere with 5 % CO_2_.

**Cell luciferase assay**: HCT 116‐Luc and MCF‐7‐Luc cells were seeded at a density of 3000 cells per well (200 μL of their corresponding medium) in 96‐well white plate, PS, F‐bottom, μCLEAR® (greiner bio‐one, Germany). Twenty‐four hours after, the cell medium was aspirated and cells were incubated with 100 μL of fresh medium containing DCP/siLuc complex formulations at the N/P ratio of 20 with the siLuc concentrations of 50, 100 and 200 nM at 37 °C for 4 h. Thereafter, 25 μL of 20 % serum containing medium was added to reach the concentration of 10 % serum and a final volume of 125 μL. Three days after transfection, expression of luciferase was assessed by addition of luciferin (10^−3^ M, final concentration) into culture medium. After 10 min, living cell luminescence was measured using a plate reader CLARIOstar® (BMG Labtech, Ortenberg, Germany) at 562 nm. The percentage of luminescence of treated cells was calculated by using the control cell (untreated) as 100 %. Each assay was repeated three times. Luciferase activity was normalized in accordance to the total number of living cells in each sample as determined by the MTT assay (see details in next paragraph).

**Cell viability (MTT) assay**: MTT (4,5‐dimethylthiazol‐2‐yl)‐2,5‐diphenyltetrazolium bromide) assay was performed to evaluate the cell viability.^[26]^ Briefly, 5000 cells were seeded into a 96 multi‐well plate in 200 μL complete culture medium. Twenty‐four hours after seeding, cells were treated with DCP/siLuc for 72 h as described in the section of “cell luciferase assay”. Cells treated with the vehicle were considered as a control. After this incubation, cells were treated for 4 h with 0.5 mg.mL^−1^ of MTT in media. The MTT/media solution was then removed and the precipitated crystals were dissolved in EtOH/DMSO (1 : 1). After 20 min of shacking, the solution absorbance (A) was read at 540 nm. The percentage of viable cells was calculated according to the following equation: %viability=A_measured_/A_control_*100.

**Statistical analysis**: Data are presented as the mean ± standard error of the mean (SEM). Statistical analysis was performed using GraphPad Prism. The comparison between groups was analyzed with Student's t‐test. Differences were considered statistically significant when p values were less than 0.05 (p<0.05). The level of significance was defined at *p<0.05, **p<0.005, and ***p<0.0005.

## Conflict of interest

The authors declare no conflict of interest.

1

## Supporting information

As a service to our authors and readers, this journal provides supporting information supplied by the authors. Such materials are peer reviewed and may be re‐organized for online delivery, but are not copy‐edited or typeset. Technical support issues arising from supporting information (other than missing files) should be addressed to the authors.

Supporting Information

## Data Availability

The data that support the findings of this study are available in the supplementary material of this article.

## References

[1] R. K. McGinty , S. Tan , Chem. Rev. 2015, 115, 2255–2273. 25495456 10.1021/cr500373h PMC4378457

[2] C. D. Allis , T. Jenuwein , Nat. Rev. Genet. 2016, 17, 487–500. 27346641 10.1038/nrg.2016.59

[3] R. Ni , J. L. Zhou , N. Hossain , Y. Chau , Adv. Drug Delivery Rev. 2016, 106, 3–26. 10.1016/j.addr.2016.07.005 27473931

[4a] K. Paunovska , D. Loughrey , J. E. Dahlman , Nat. Rev. Genet. 2022, 23, 265–280; 34983972 10.1038/s41576-021-00439-4 PMC8724758

[4b] C. O. Franck , L. Fanslau , A. B. Popov , P. Tyagi , L. Fruk , Angew. Chem. Int. Ed. 2021, 60, 13225–13243. 10.1002/anie.202010282 PMC8247987 32893932

[5a] S. Ulrich , Acc. Chem. Res. 2019, 52, 510–519; 30676745 10.1021/acs.accounts.8b00591

[5b] E. Wagner , Acc. Chem. Res. 2012, 45, 1005–1013. 22191535 10.1021/ar2002232

[6] X. X. Liu , J. H. Zhou , T. Z. Yu , C. Chen , Q. Cheng , K. Sengupta , Y. Y. Huang , H. T. Li , C. Liu , Y. Wang , P. Posocco , M. H. Wang , Q. Cui , S. Giorgio , M. Fermeglia , F. Q. Qu , S. Pricl , Y. H. Shi , Z. C. Liang , P. Rocchi , J. J. Rossi , L. Peng , Angew. Chem. Int. Ed. 2014, 53, 11822–11827; 10.1002/anie.201406764 PMC4485617 25219970

[7] P. Evenou , J. Rossignol , G. Pembouong , A. Gothland , D. Colesnic , R. Barbeyron , S. Rudiuk , A. G. Marcelin , M. Menand , D. Baigl , V. Calvez , L. Bouteiller , M. Sollogoub , Angew. Chem. Int. Ed. 2018, 57, 7753–7758; 10.1002/anie.201802550 29693753

[8a] A. Kohata , P. K. Hashim , K. Okuro , T. Aida , J. Am. Chem. Soc. 2019, 141, 2862–2866; 30724083 10.1021/jacs.8b12501

[8b] J. Zhou , L. Sun , L. P. Wang , Y. C. Liu , J. Y. Li , J. Y. Li , J. Li , H. H. Yang , Angew. Chem. Int. Ed. 2019, 58, 5236–5240; 10.1002/anie.201813665 30809927

[8c] P. K. Hashim , K. Okuro , S. Sasaki , Y. Hoashi , T. Aida , J. Am. Chem. Soc. 2015, 137, 15608–15611; 26648391 10.1021/jacs.5b08948

[8d] E. K. Bang , M. Lista , G. Sforazzini , N. Sakai , S. Matile , Chem. Sci. 2012, 3, 1752–1763.

[9a] C. Xu , X. W. Guan , L. Lin , Q. Wang , B. Gao , S. H. Zhang , Y. H. Li , H. Y. Tian , ACS Biomater. Sci. Eng. 2018, 4, 193–199; 33418689 10.1021/acsbiomaterials.7b00869

[9b] E. Bartolami , Y. Bessin , V. Gervais , P. Dumy , S. Ulrich , Angew. Chem. Int. Ed. 2015, 54, 10183–10187; 10.1002/anie.201504047 26177835

[9c] E. Bartolami , Y. Bessin , N. Bettache , M. Gary-Bobo , M. Garcia , P. Dumy , S. Ulrich , Org. Biomol. Chem. 2015, 13, 9427–9438; 26248061 10.1039/c5ob01404b

[9d] D. B. Rozema , D. L. Lewis , D. H. Wakefield , S. C. Wong , J. J. Klein , P. L. Roesch , S. L. Bertin , T. W. Reppen , Q. Chu , A. V. Blokhin , J. E. Hagstrom , J. A. Wolff , Proc. Natl. Acad. Sci. USA 2007, 104, 12982–12987. 17652171 10.1073/pnas.0703778104 PMC1941806

[10a] N. Laroui , M. Coste , D. Su , L. M. A. Ali , Y. Bessin , M. Barboiu , M. Gary-Bobo , N. Bettache , S. Ulrich , Angew. Chem. Int. Ed. 2021, 60, 5783–5787; 10.1002/anie.202014066 33289957

[10b] C. Bouillon , Y. Bessin , F. Poncet , M. Gary-Bobo , P. Dumy , M. Barboiu , N. Bettache , S. Ulrich , J. Mater. Chem. B 2018, 6, 7239–7246; 32254636 10.1039/c8tb01278d

[10c] C. Bouillon , D. Paolantoni , J. C. Rote , Y. Bessin , L. W. Peterson , P. Dumy , S. Ulrich , Chem. Eur. J. 2014, 20, 14705–14714. 25251569 10.1002/chem.201403695

[11] M. Surin , S. Ulrich , ChemistryOpen 2020, 9, 480–498. 32328404 10.1002/open.202000013 PMC7175023

[12] J. K. Strauss , L. J. Maher , Science 1994, 266, 1829–1834. 7997878 10.1126/science.7997878

[13] A. Estévez-Torres , D. Baigl , Soft Matter 2011, 7, 6746–6756.

[14a] V. B. Teif , K. Bohinc , Prog. Biophys. Mol. Biol. 2011, 105, 208–222; 20638406 10.1016/j.pbiomolbio.2010.07.002

[14b] V. A. Bloomfield , Biopolymers 1997, 44, 269–282. 9591479 10.1002/(SICI)1097-0282(1997)44:3<269::AID-BIP6>3.0.CO;2-T

[15] N. Pastor , Biophys. J. 2005, 88, 3262–3275. 15749779 10.1529/biophysj.104.058339 PMC1305475

[16a] I. Lostalé-Seijo , J. Montenegro , Nat. Chem. Rev. 2018, 2, 258–277;

[16b] I. Louzao , R. Garcia-Fandino , J. Montenegro , J. Mater. Chem. B 2017, 5, 4426–4434; 32263970 10.1039/c7tb00179g

[16c] M. Nguyen , J. L. Stigliani , G. Pratviel , C. Bonduelle , Chem. Commun. 2017, 53, 7501–7504; 10.1039/c7cc03472e 28628166

[16d] P. Boisguerin , S. Deshayes , M. J. Gait , L. O′Donovan , C. Godfrey , C. A. Betts , M. J. A. Wood , B. Lebleu , Adv. Drug Delivery Rev. 2015, 87, 52–67; 10.1016/j.addr.2015.02.008 PMC7102600 25747758

[16e] J. Hoyer , I. Neundorf , Acc. Chem. Res. 2012, 45, 1048–1056. 22455499 10.1021/ar2002304

[17] CtDNA was chosen as a commercially-available source of long double-stranded DNA.

[18] E. W. Gerner , F. L. Meyskens , Nat. Rev. Cancer 2004, 4, 781–792. 15510159 10.1038/nrc1454

[19] M. A. Kostiainen , J. G. Hardy , D. K. Smith , Angew. Chem. Int. Ed. 2005, 44, 2556–2559; 10.1002/anie.200500066 15782377

[20a] D. K. Kölmel , E. T. Kool , Chem. Rev. 2017, 117, 10358–10376; 28640998 10.1021/acs.chemrev.7b00090 PMC5580355

[20b] S. Ulrich , D. Boturyn , A. Marra , O. Renaudet , P. Dumy , Chem. Eur. J. 2014, 20, 34–41. 24302514 10.1002/chem.201302426

[21] For a recent review on polyacylhydrazones, see K. Caillaud , C. Ladaviere , Macromol. Chem. Phys. 2022, 2200064.

[22a] R. Kumar , N. Le , Z. Tan , M. E. Brown , S. Jiang , T. M. Reineke , ACS Nano 2020, 14, 17626–17639; 33225680 10.1021/acsnano.0c08549

[22b] M. R. Molla , P. A. Levkin , Adv. Mater. 2016, 28, 1159–1175; 26608939 10.1002/adma.201502888

[22c] A. C. Rinkenauer , A. Vollrath , A. Schallon , L. Tauhardt , K. Kempe , S. Schubert , D. Fischer , U. S. Schubert , ACS Comb. Sci. 2013, 15, 475–482. 23886244 10.1021/co400025u

[23] S. Foillard , M. O. Rasmussen , J. Razkin , D. Boturyn , P. Dumy , J. Org. Chem. 2008, 73, 983–991. 18173281 10.1021/jo701628k

[24a] V. Dulery , O. Renaudet , P. Dumy , Tetrahedron 2007, 63, 11952–11958;

[24b] M. Kurono , A. Shimomura , M. Isobe , Tetrahedron 2004, 60, 1773–1780.

[25] Y. C. Huang , C. C. Chen , S. J. Li , S. Gao , J. Shi , Y. M. Li , Tetrahedron 2014, 70, 2951–2955.

[26] P. Kumar , A. Nagarajan , P. D. Uchil , Cold Spring Harbor Protoc. 2018, 6, 10.1101/pdb.prot095505. 29858336

