# Supplementary material for: Structure‐Activity Relationships in Nucleic‐Acid‐Templated Vectors Based on Peptidic Dynamic Covalent Polymers
Source: Chemistry. 2022 Dec 12;29(7):e202202921. doi: 10.1002/chem.202202921 (PMC10108046; doi:10.1002/chem.202202921)
Supplement: Supplementary file 1 — Supporting Information [file CHEM-29-0-s001.pdf]

Chemistry–A European Journal

Supporting Information

Structure-Activity Relationships in Nucleic-Acid-Templated Vectors Based on Peptidic Dynamic Covalent Polymers

Dan-Dan Su, Lamiaa M. A. Ali, Maëva Coste, Nabila Laroui, Yannick Bessin, Mihail Barboiu,* Nadir Bettache,* and Sébastien Ulrich*

1. Synthesis of bisaldehyde peptides **BisAldn**.....2
2. Synthesis of N-aminooxy, C-hydrazide peptides **OxArg$_n$Hyd**.....14
3. Complexation of ctDNA monitored by the fluorescence displacement assay.....20
4. Gel retardation assay for pDNA complexation.....20
5. Gel retardation assay for siRNA complexation.....22
6. Knock-down of luciferase activity by siRNA delivery.....23
7. References.....24

1. Synthesis of bisaldehyde peptides BisAldn

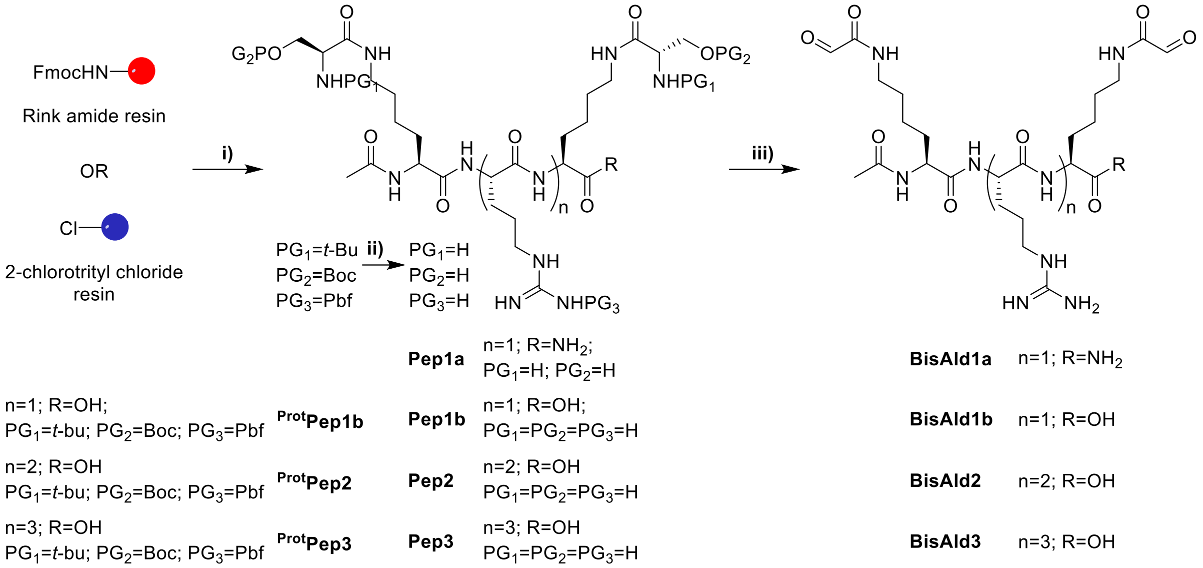

Scheme S1: General synthetic scheme for the preparation of bisaldehyde peptides. i) SPPS using Fmoc-*L*-Lys[Boc-*L*-Ser(OtBu)]OH, ii) deprotection in TFA/TIS/H_2O 95/2.5/2.5; iii) oxidative cleavage with $NaIO_4$. Amide C-termini (R=NH_2) obtained when starting from the Rink amide resin, carboxylic acid C-termini (R=OH) obtained when starting from the 2-chlorotrityl chloride resin. Boc: *tert*-butyloxycarbonyl.

General procedure. The solid phase peptide synthesis was carried out manually in a Fmoc strategy using Fmov-*L*-Arg(Pbf)-OH and Fmoc-*L*-Lys[Boc-*L*-Ser(*t*-Bu)]-OH. The following sequence was used:

Deprotection of the AmphiSphere Rink amide resin:
1) Resin deprotection: piperidine/DMF (2/8) at r.t. for 1 min, twice
2) The resin was then directly engaged in SPPS (see procedure below)

Loading of the 2-chlorotrityl resin:
1) Resin deprotection: piperidine/DMF (2/8) at r.t. for 1 min, twice
2) Resin loading: the 2-chlorotrityl resin (loading 0.2 mmol/g) was loaded with Fmoc-*L*-Lys[Boc-*L*-Ser(t-Bu)]-OH. To a stirred suspension of 2-chlorotrityl resin (5.25 g, 1.6 mmol Cl/g) in NMP/DMSO was added DIEA (7.35 mL, 42.2 mmol). Fmoc-*L*-Lys[Boc-*L*-Ser(t-Bu)]-OH (10.42 g, 17.03 mmol) was added and the reaction mixture was stirred for 48 h at room temperature. The reaction mixture was filtered and capping was carried out using MeOH for 30 min, twice. Then the resin was washed with DMSO (3x), CH_2Cl_2 (2x), isopropanol (1x), CH_2Cl_2 (2x) and Et_2O (3x) and dried in vacuo.

Peptide synthesis/elongation:

3) Coupling procedure: Fmoc-*L*-Arg(Pbf)-OH (5 eq., 0.6 M), HATU (5 eq., 0.2 M), DIEA (10 eq.), stirred at r.t. in DMF for 30 min, twice; or Fmoc-*L*-Lys[Boc-*L*-Ser(t-Bu)]-OH (3 eq., 0.6 M), HATU (3 eq., 0.2 M), DIEA (6 eq.), stirred at r.t. in DMF for 30 min, twice

3) Fmoc deprotection: piperidine/DMF (2/8) at r.t. for 1 min, twice

4) Acetylation: Ac_2O (150 eq.)/DCM, 1/1 (v/v), at r.t. for 3 min, twice

One-pot cleavage and deprotection from Rink amide resin:

5) TFA/TIS/H_2O (95/2.5/2.5) at r.t. for 12 h, then precipitation in diethyl ether. The precipitates were freeze-dried to give white solids.

Mild cleavage followed by deprotection from 2-chlorotrityl resin:

5) Mild cleavage: TFA/DCM (1/99) at r.t. for 5 minutes, 4 times. Filtration and neutralization of the solution using MeOH/Pyridine 8/2. The peptides were purified by preparative reverse-phase HPLC using the following gradient of eluents A and B: 0 min, 95% A, 5% B; 5 min, 95% A, 5% B; 45 min, 5% A, 95% B; 50 min, 100% B.

6) Deprotection: TFA/TIS/H_2O (95/2.5/2.5) at r.t. for 12 h, then precipitation in diethyl ether. The precipitates were freeze-dried to give white solids.

Oxidative cleavage:

$NaIO_4$ (10 eq.) was added onto a 10 mM solution of peptides in water, at r.t. for 2 h, then precipitated in diethyl ether. Purification was carried out by using Size Exclusion Chromatography (Sephadex G-10), before freeze-drying.

The final bisaldehyde peptides were all titrated by ^1H NMR (D_2O) using *tert*-butanol as internal reference in order to determine their exact concentration. For this, the compound was solubilized in D_2O and *tert*-butyl alcohol was added (20 µL, 31 mM) in the NMR tube (total volume of 600 µL). ^1H NMR was recorded and the relative peak integration was used to calculate the exact concentration of the compound.

^{Prot}Pep1b. Yield: 13.4%, 58.4 mg, 48.2 µmol. HPLC t_R: 4.867 min. ESI-MS m/z calcd for $[C_{57}H_{98}N_{10}O_{16}S+H]^+$ 1211.70, found 1211.70.

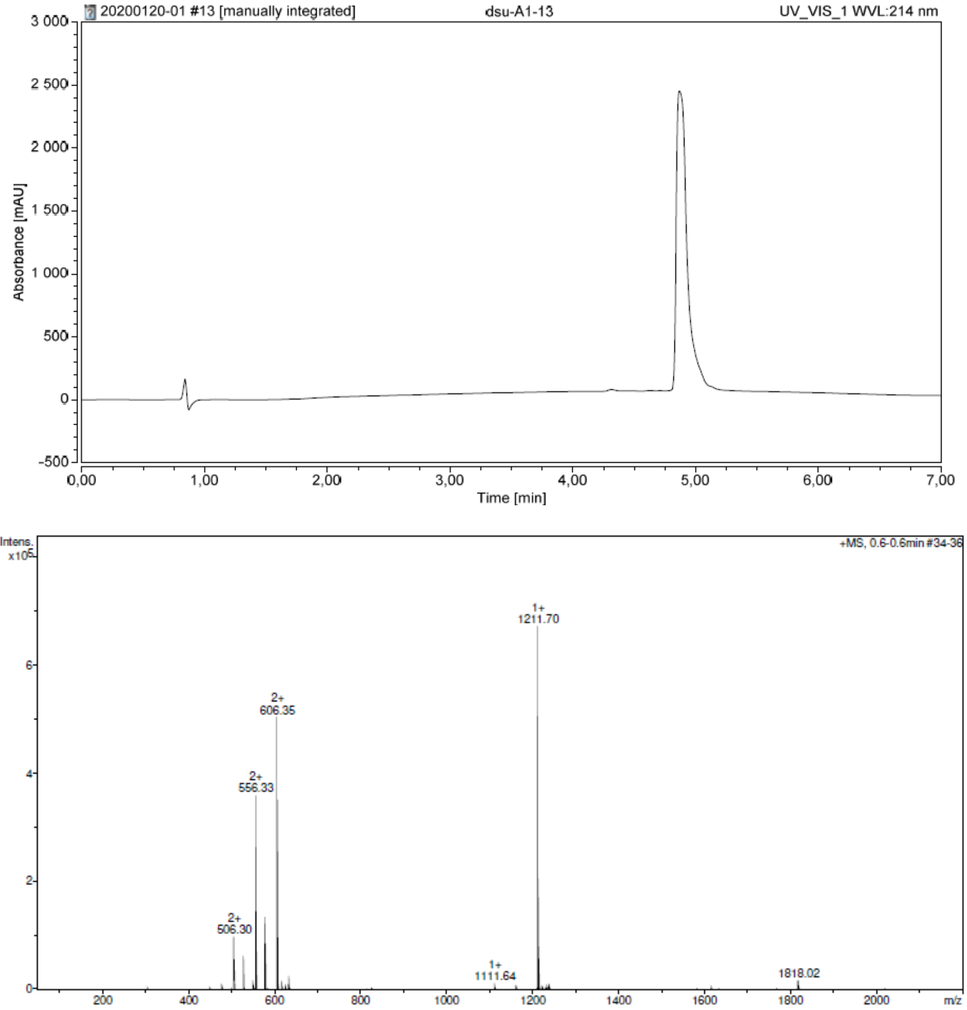

Figure S1: LC/MS analysis: UV chromatogram (top) and MS spectrum (bottom).

^{Prot}Pep2. Yield: 32%, 186 mg, 115 µmol. HPLC t_R: 5.307 min. ESI-MS m/z calcd for $[C_{76}H_{126}N_{14}O_{20}S_2+2H]^{2+}$ 810.44, found 810.44.

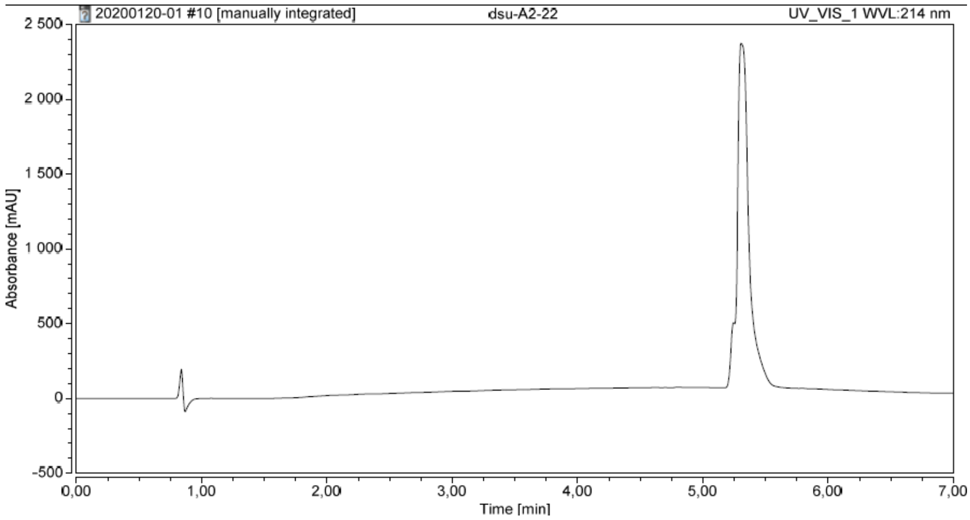

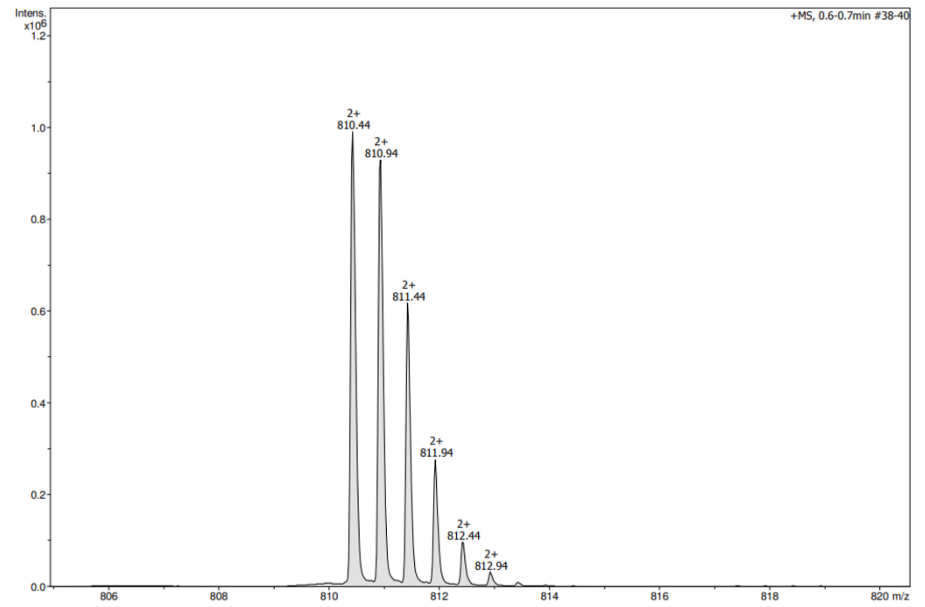

Figure S2: LC/MS analysis: UV chromatogram (top) and MS spectrum (bottom).

^{Prot}Pep3. Yield: 8.2%, 29.8 mg, 29.4 µmol. HPLC t_R: 5.447 min. ESI-MS m/z calcd for $[C_{95}H_{154}N_{18}O_{24}S_3+2H]^{2+}$ 1014.53, found 1014.65.

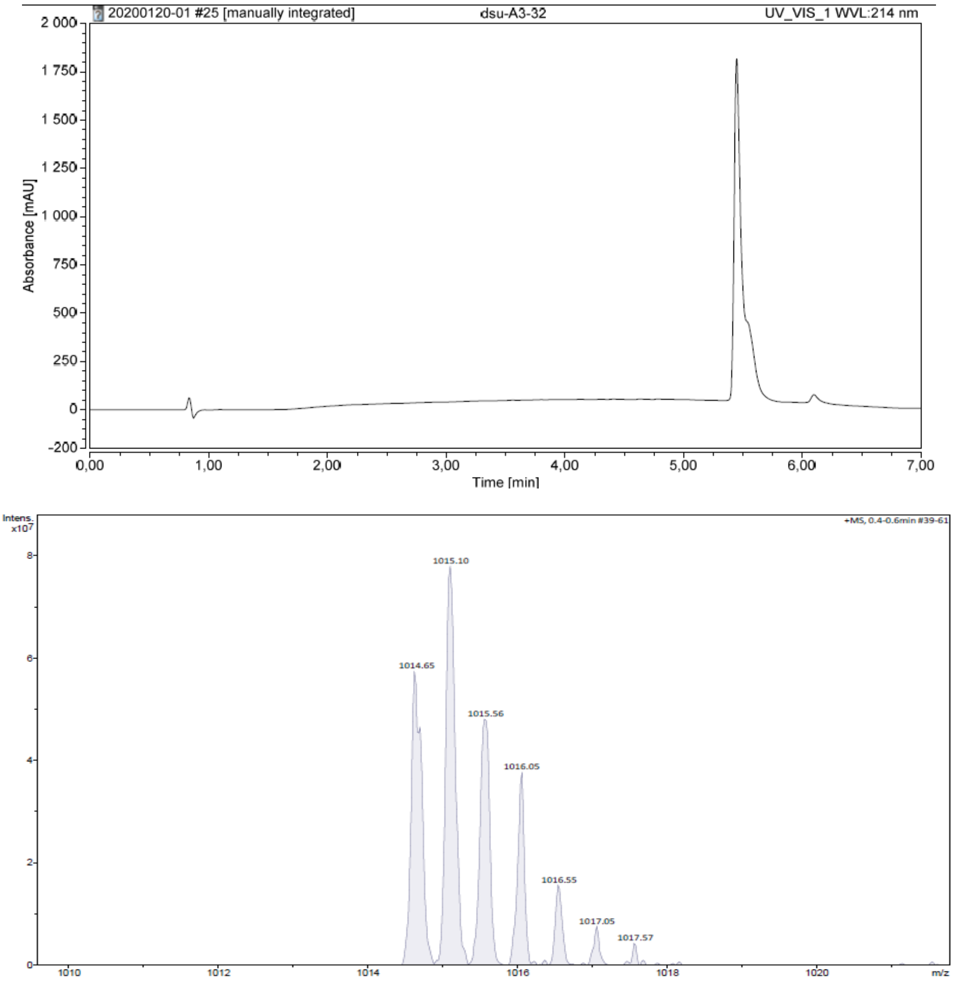

Figure S3: LC/MS analysis: UV chromatogram (top) and MS spectrum (bottom).

Pep1a. The peptide was purified by preparative reverse-phase HPLC [HPLC 2] using the following gradient of eluents A and B: 0 min, 100% A; 5 min, 100% A; 50 min, 50% A, 50% B. Yield: 46%. LC/MS t_R: 0.564 min; MS calcd for m/z $[C_{26}H_{51}N_{11}O_8+H]^+$ 646.39, found 646.45.

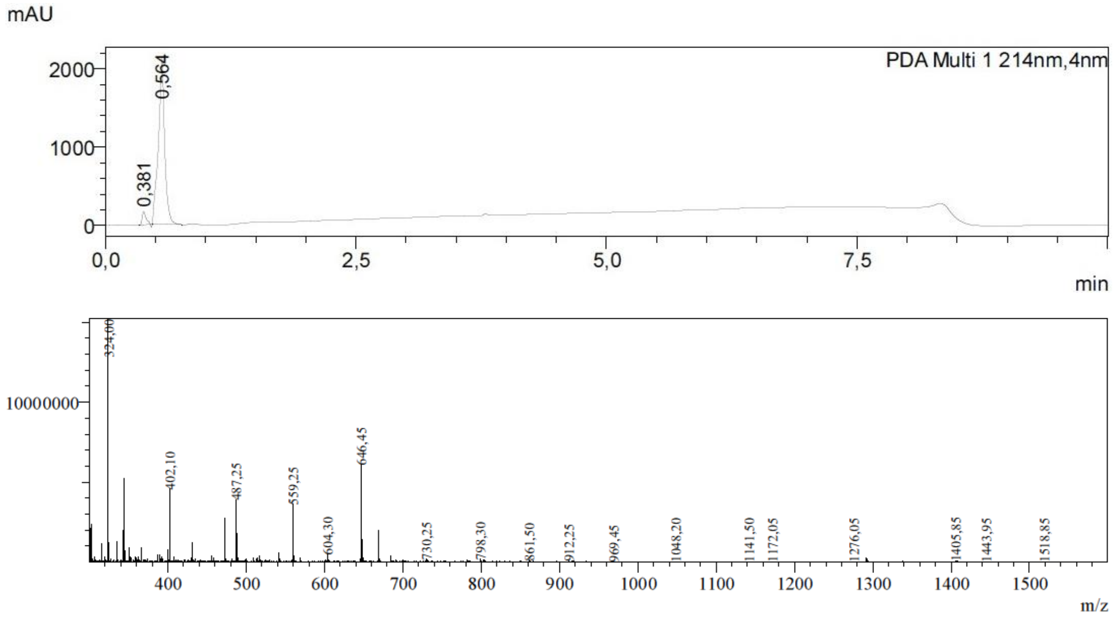

Figure S4: LC/MS analysis: UV chromatogram (top) and MS spectrum (bottom).

Pep1b. Yield: 7.8%, 31.2 mg, 48.3 µmol. ^1H NMR (400 MHz, D_2O): δ= 4.39-4.22 (m, 3H), 4.10 (t, ^3J = 5.0, 2H), 4.02-3.92 (m, 4H), 3.29-3.21 (m, 6H), 2.04 (s, 3H), 1.88-1.76 (m, 6H), 1.69-1.57 (m, 6H), 1.45-1.33 (m, 4H). ESI-MS m/z calcd for $[C_{26}H_{50}N_{10}O_9+H]^+$ 647.38, found 647.4.

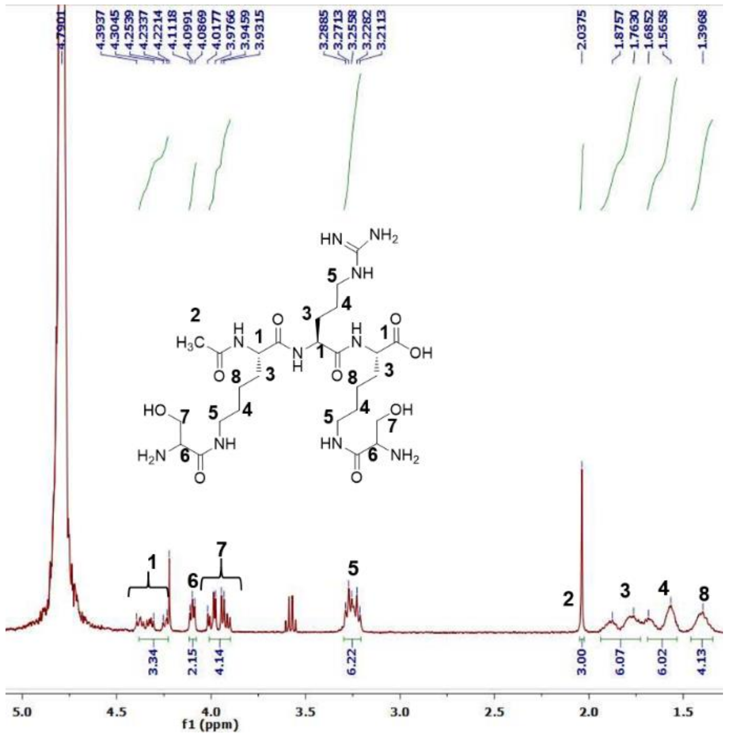

Figure S5: ^1H NMR (400 MHz, D_2O) spectrum.

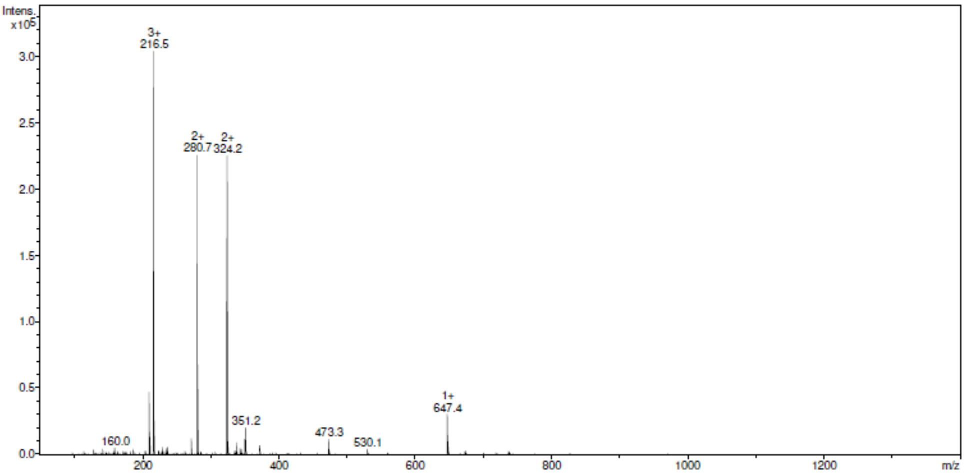

Figure S6: ESI-MS spectrum.

Pep2. Yield: 17.6%, 50.8 mg, 63 μmol. ^{1}H NMR (400 MHz, D_2O): δ= 4.36-4.22 (m, 4H), 4.10 (t, ^{3}J = 5.0, 2H), 4.02-3.92 (m, 4H), 3.29-3.20 (m, 8H), 2.04 (s, 3H), 1.87-1.78 (m, 8H), 1.68-1.57 (m, 8H), 1.45-1.35 (m, 4H). ESI-MS m/z calcd for $[C_{32}H_{62}N_{14}O_{10}+H]^{+}$ 803.49, found 803.5.

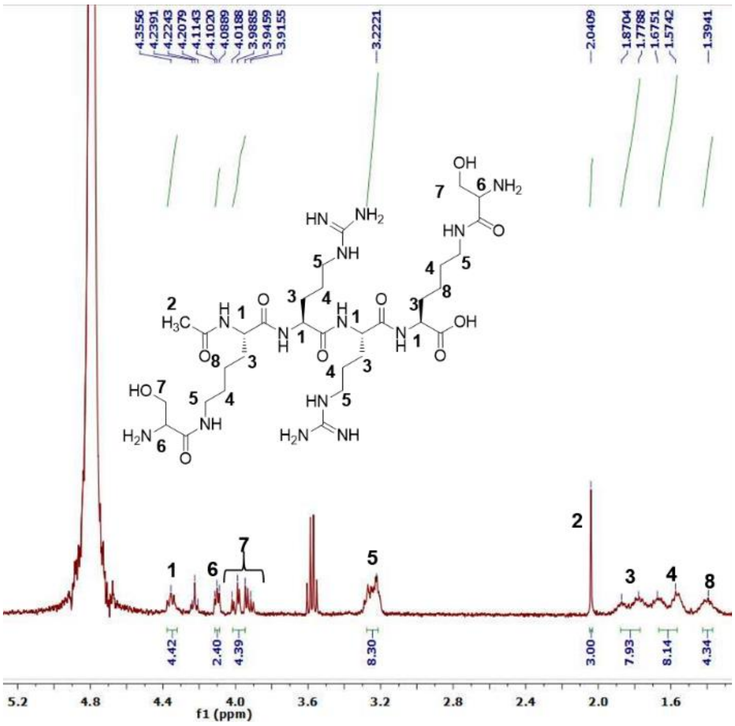

Figure S7: ^{1}H NMR (400 MHz, D_2O) spectrum.

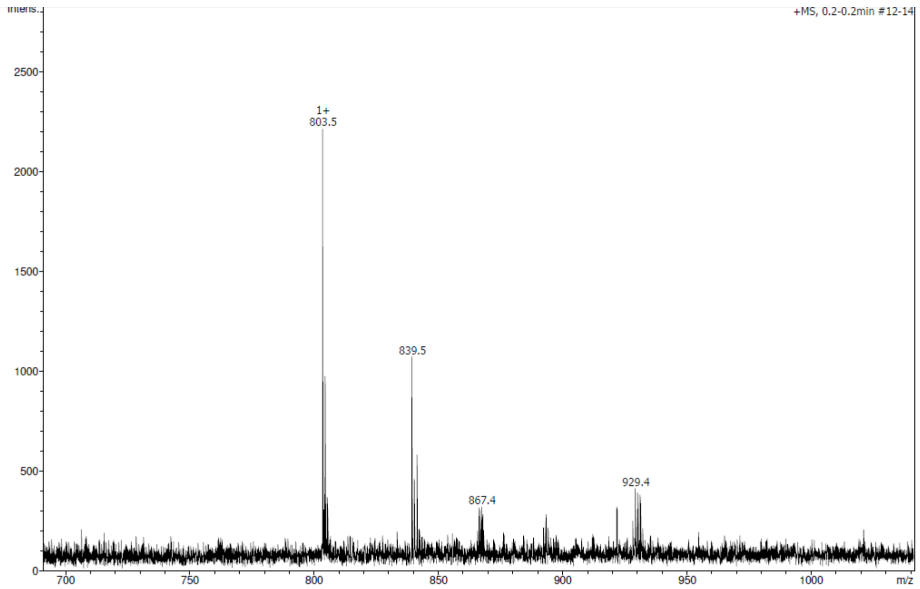

Figure S8: ESI-MS spectrum.

Pep3. Yield: 4.8%, 16.6 mg, 17.3 µmol. ^{1}H NMR (400 MHz, D_2O): δ= 4.36-4.21 (m, 5H), 4.11 (s, 2H), 4.02-3.91 (m, 4H), 3.30-3.18 (m, 10H), 2.05 (s, 3H), 1.84-1.78 (m, 10H), 1.67-1.57 (m, 10H), 1.45-1.34 (m, 4H). ESI-MS m/z calcd for $[C_{38}H_{74}N_{18}O_{11}+H]^+$ 959.5863, found 959.5871.

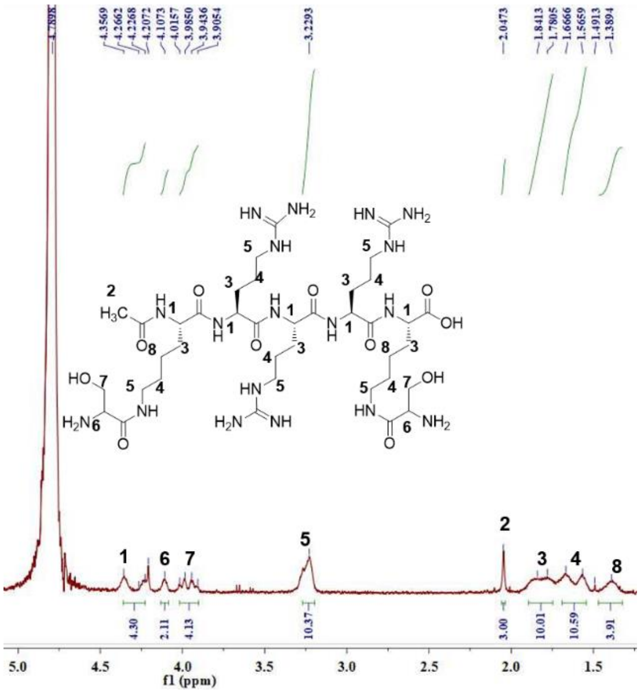

Figure S9: ^{1}H NMR (400 MHz, D_2O) spectrum.

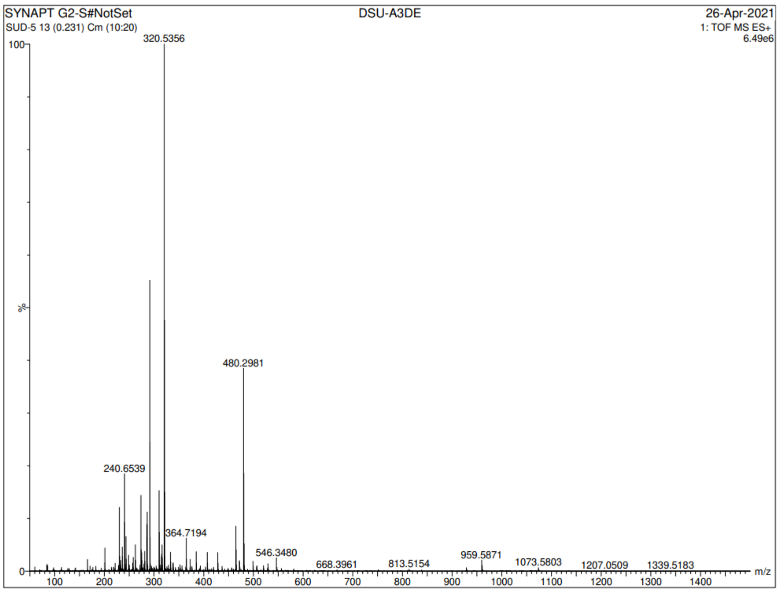

Figure S10: HR-ESI-MS spectrum.

BisAld1a. Oxidative cleavage was carried out using the general procedure on 74 mg of **Pep1a**. The desired product was obtained in 32% yield. ^{1}H NMR (400 MHz, D_2O): δ = 4.28-4.24 (m, 1H, CHα), 4.20-4.17 (m, 1H, CHα), 4.15-4.11 (m, 1H, CHα), 3.21-3.17 (m, 4H, CH_2), 3.15-3.12 (m, 2H, CH_2), 1.94 (s, 3H, CH_3), 1.77-1.63 (m, 6H, CH_2), 1.56-1.46 (m, 6H, CH_2), 1.33-1.25 (m, 4H, CH_2). LC/MS: t_R 0.6-1.3 min; MS calcd for m/z $[C_{24}H_{41}N_9O_8+H]^+$ = 584.31, found $[C_{24}H_{41}N_9O_8+2H_2O+H]^+$ = 620.34; $[C_{24}H_{41}N_9O_8+2H]^{2+}$ = 292.69.

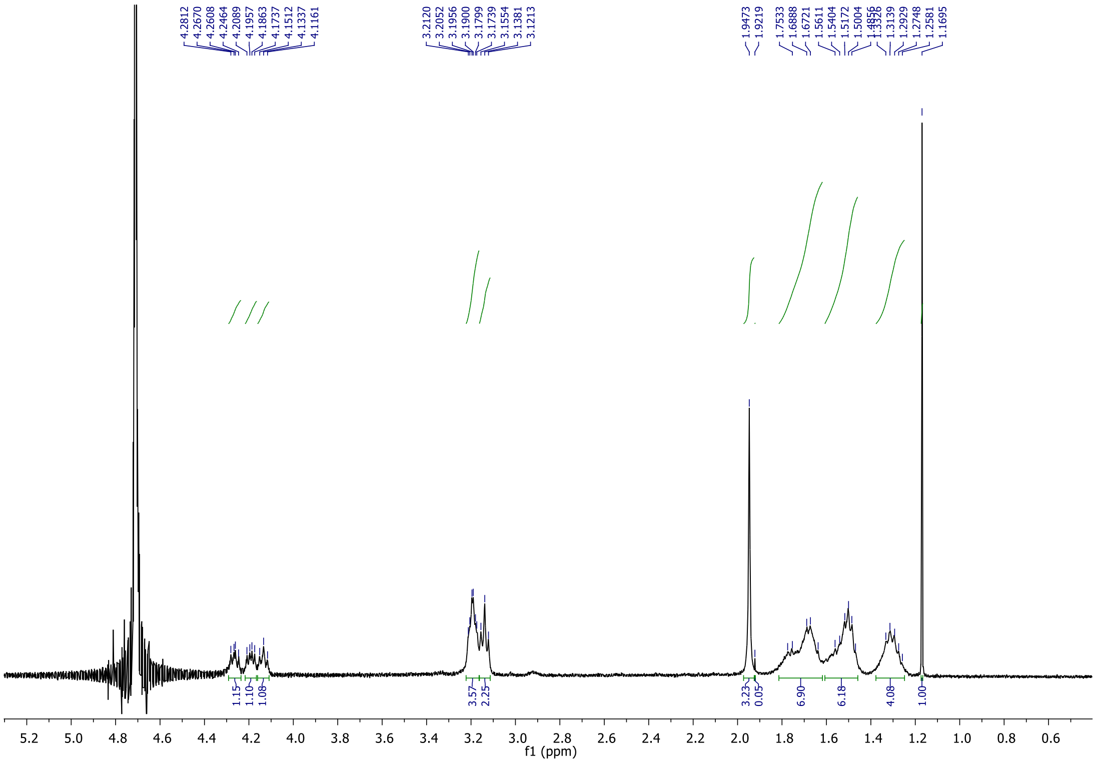

Figure S11: ^{1}H NMR (400 MHz, D_2O) spectrum. The sharp singlet at 1.69 ppm corresponds to the *tert*-BuOH reference.

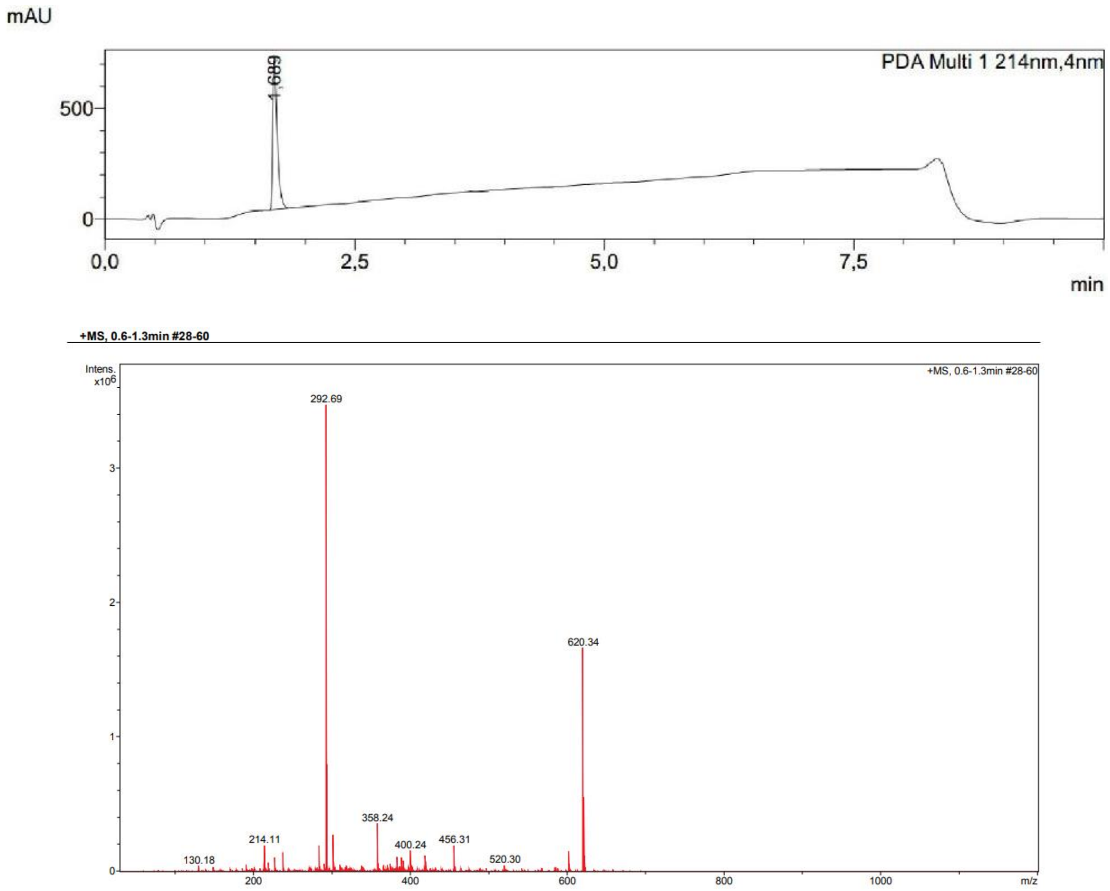

Figure S12: HPLC analysis (UV chromatogram, top), and ESI-MS spectrum (bottom).

BisAld1b. Yield: 5.9%, 21.1 µmol. ^{1}H NMR (400 MHz, D_2O): δ= 4.40-4.23 (m, 3H), 4.08 (s, 2H), 3.31-3.24 (m, 6H), 2.05 (s, 3H), 1.91-1.78 (m, 6H), 1.69-1.58 (m, 6H), 1.45-1.37 (m, 4H). ESI-MS m/z calcd for $[C_{24}H_{40}N_8O_9+H]^+$ 585.30, found 585.15.

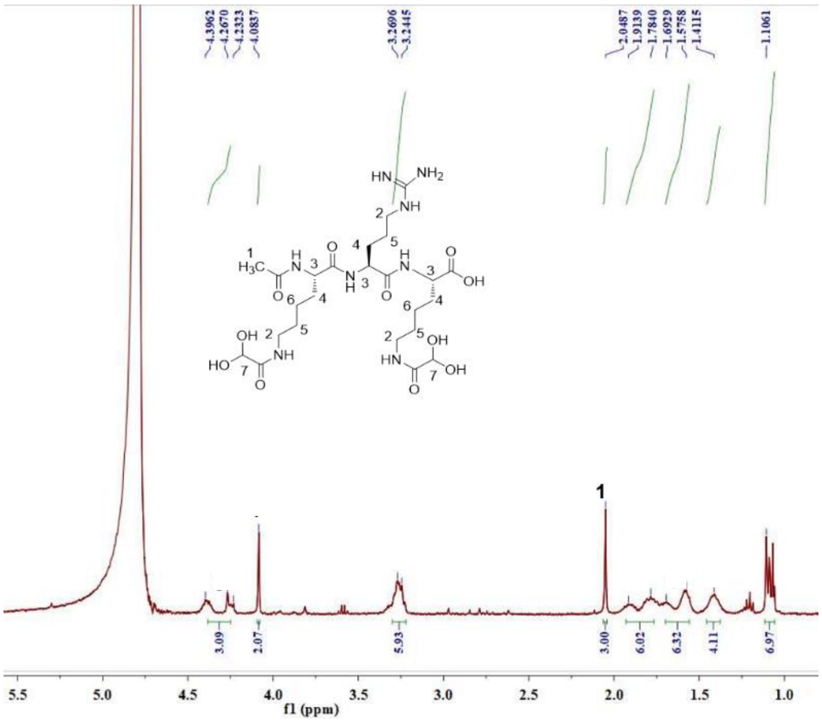

Figure S13: ^{1}H NMR (400 MHz, D_2O) spectrum.

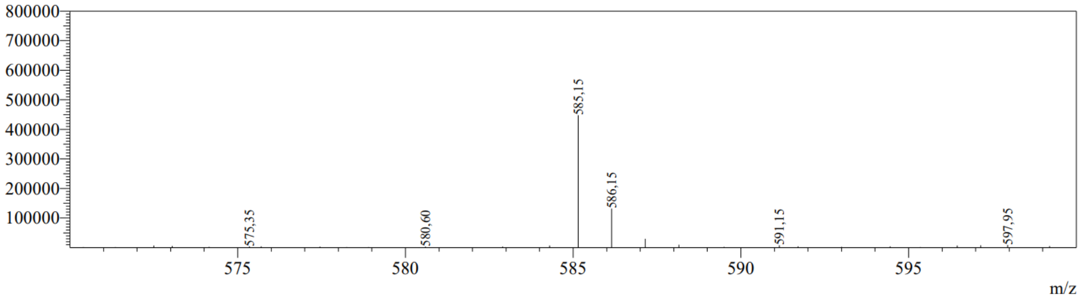

Figure S14: ESI-MS spectrum.

BisAld2. Yield: 3.9%, 13.9 µmol. ^{1}H NMR (400 MHz, D_2O): δ= 4.37-4.23 (m, 4H), 4.08 (s, 2H), 3.26-3.25 (m, 8H), 2.05 (s, 3H), 1.91-1.78 (m, 6H), 1.66-1.58 (m, 6H), 1.44-1.37 (m, 4H). ESI-MS m/z calcd for $[C_{30}H_{52}N_{12}O_{10}+H+NH4]^{2+}$ 379.72, found 380.15.

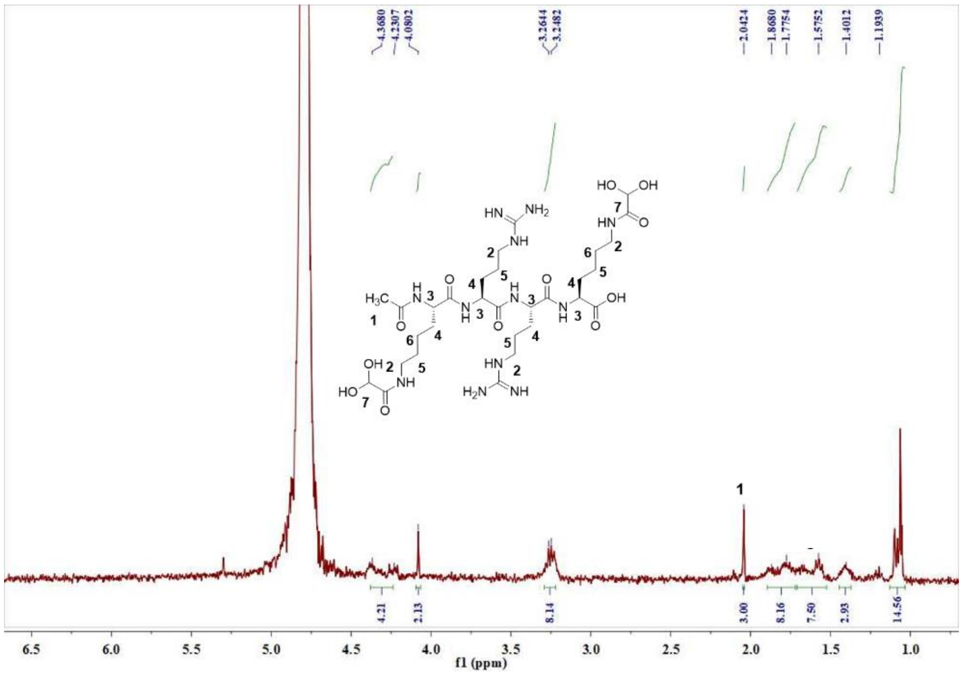

Figure S15: ^1H NMR (400 MHz, D_2O) spectrum.

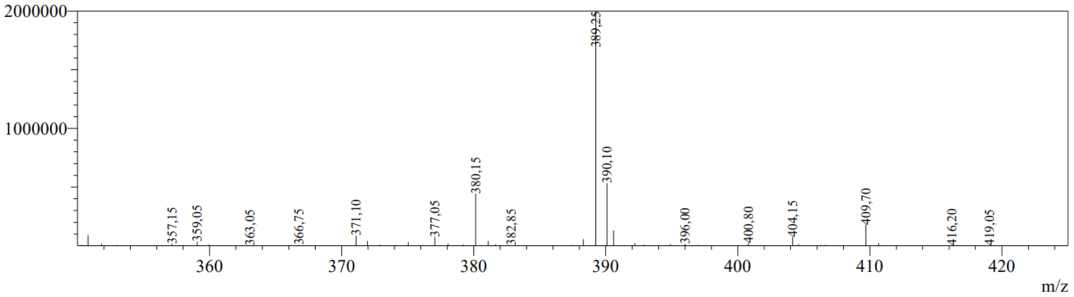

Figure S16: ESI-MS spectrum.

BisAld3. Yield: 0.6%, 2.3 µmol. ^1H NMR (400 MHz, D_2O): δ= 4.39-4.34 (m, 5H), 4.08 (s, 2H), 3.28-3.19 (m, 10H), 2.04 (s, 3H), 1.87-1.79 (m, 10H), 1.66-1.58 (m, 10H), 1.43-1.37 (m, 4H). ESI-MS m/z calcd for $[C_{36}H_{64}N_{16}O_{11}+H]^+$ 897.5013, found 897.5020.

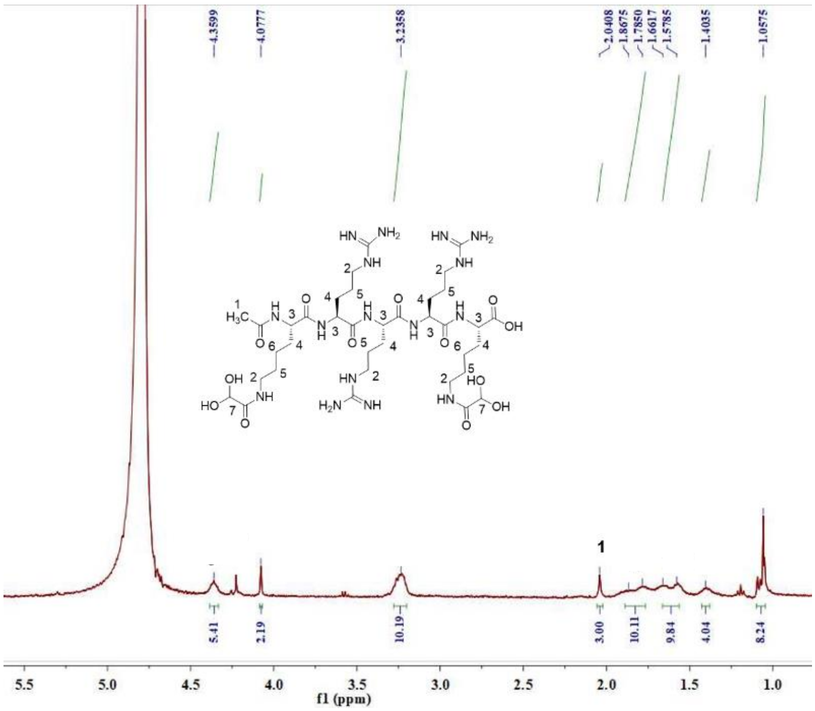

Figure S17: ^{1}H NMR (400 MHz, D_2O) spectrum.

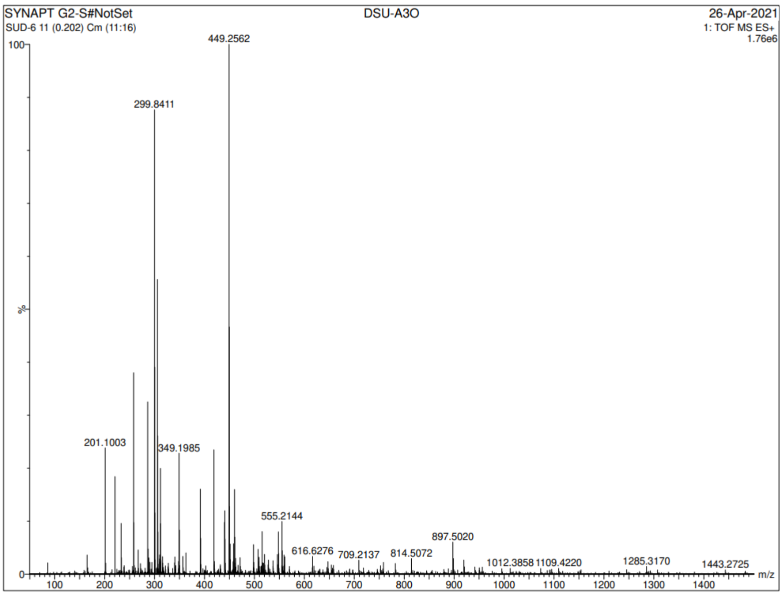

Figure S18: HR-ESI-MS spectrum.

2. Synthesis of N-aminooxy, C-hydrazide peptides $OxArg_nHyd$

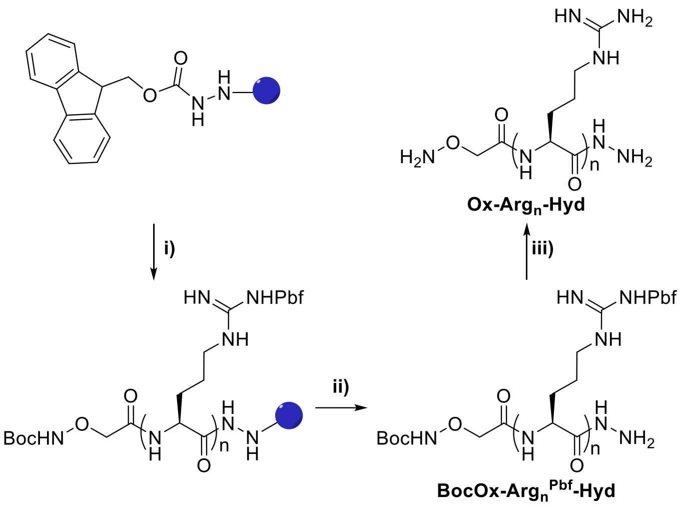

Scheme S2: General synthetic scheme for the preparation of N-aminooxy, C-hydrazide peptides $OxArg_nHyd$. i) SPPS on Fmoc-protected hydrazine resin, terminated by a coupling with N'-Boc-aminooxyacetyl N-hydroxysuccinimide ester, ii) mild cleavage; iii) deprotection. 2-chlorotrityl chloride resin. Boc: *tert*-butyloxycarbonyl. Pbf: 2,2,4,6,7-pentamethyldlhydrobenzofuran-5-sulfonyl.

General procedure. The solid phase peptide synthesis was carried out manually in a Fmoc-protected hydrazine resin. The following sequence was used:

1) Fmoc-protected hydrazine resin deprotection: piperidine/DMF (2/8) at r.t. for 1 min, twice
2) Peptide coupling procedure: Fmoc-*L*-Arg(Pbf)-OH (3 eq.), HATU (3 eq.), DIEA (6 eq.), stirred at r.t. in DMF for 30 min, twice
3) Fmoc deprotection: piperidine/DMF (2/8) at r.t. for 1 min, twice
4) Aminooxy functionalization: N'-Boc-aminooxyacetyl N-hyroxysuccinimide ester (5 eq.), DIEA (5 eq.), stirred at r.t. in DMF for 12 h
5) Mild cleavage: TFA/DCM (1/99) at r.t. for 5 minutes, 4 times. Filtration and neutralization of the solution using MeOH/Pyridine 8/2. The peptides were purified by preparative reverse-phase HPLC using the following gradient of eluents A and B: 0 min, 80% A, 20% B; 5 min, 80% A, 20% B; 45 min, 20% A, 80% B; 50 min, 100% B.
6) Deprotection: TFA/TIS/H_2O (95/2.5/2.5) at r.t. for 12 h, then precipitation in diethyl ether. The precipitates were freeze-dried to give white solids.

The final N-aminooxy, C-hydrazide peptides were all titrated by ^1H NMR (D_2O) using *tert*-butanol as internal reference in order to determine their exact concentration. For this, the compound was solubilized in D_2O and *tert*-butyl alcohol was added (20 µL, 31 mM) in the NMR tube (total volume of 600 µL). ^1H NMR was recorded and the relative peak integration was used to calculate the exact concentration of the compound.

BocOx-Arg^{Pbf}-Hyd. Yield: 89%, 197.7 mg, 322.4 µmol. HPLC t_R: 3.96 min. ESI-MS m/z calcd for $[C_{26}H_{43}N_7O_8S+H]^+$ 614.30, found 614.30.

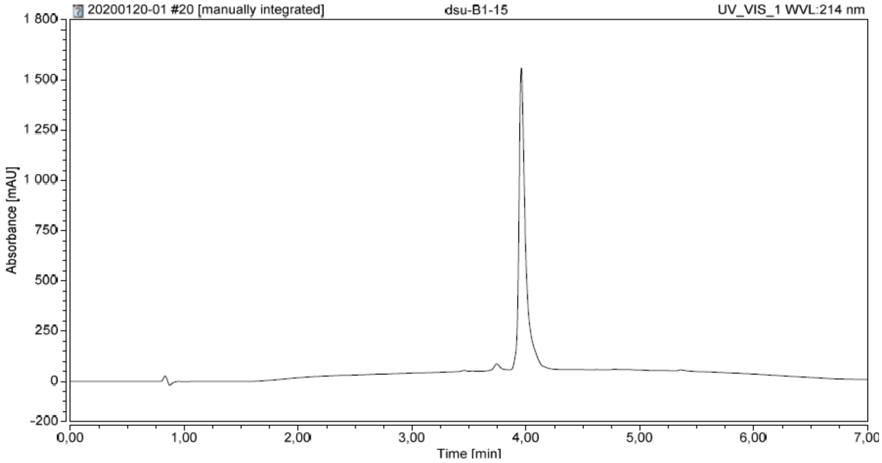

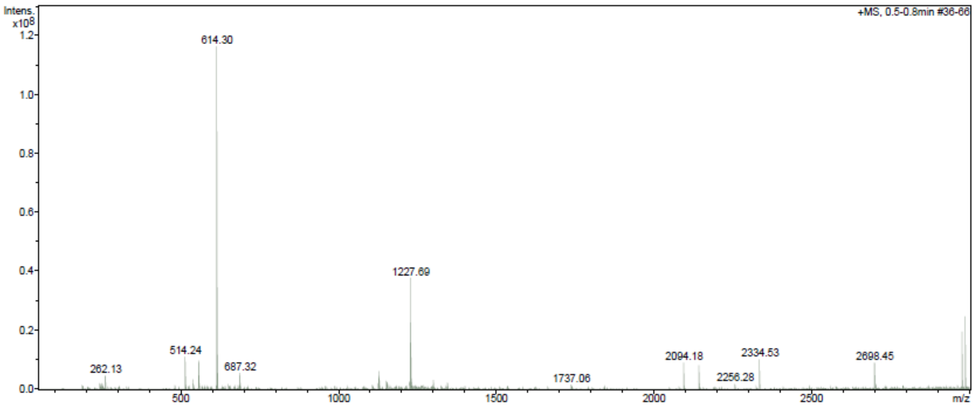

Figure S19: LC/MS analysis: UV chromatogram (top) and MS spectrum (bottom).

BocOx-Arg$_2^{Pbf}$-Hyd. Yield: 9.6%, 35.4 mg, 34.7 µmol. HPLC t_R: 4.63 min. ESI-MS m/z calcd for $[C_{45}H_{71}N_{11}O_{12}S_2+H]^+$ 1022.48, found 1022.57.

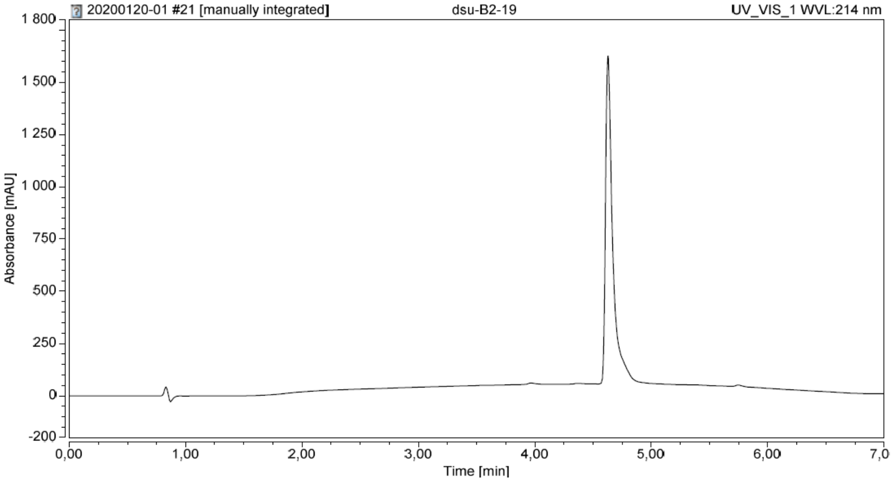

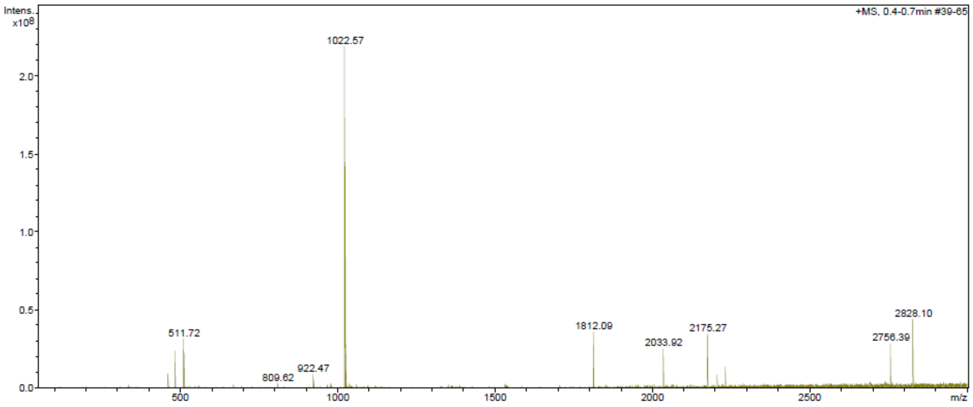

Figure S20: LC/MS analysis: UV chromatogram (top) and MS spectrum (bottom).

BocOx-Arg$_3^{Pbf}$-Hyd. Yield: 77%, 405 mg, 283 µmol. HPLC t_R: 5.12 min. ESI-MS m/z calcd for $[C_{64}H_{99}N_{15}O_{16}S_3+H]^+$ 1430.66, found 1430.66; $[C_{64}H_{99}N_{15}O_{16}S_3+2H]^{2+}$ 715.84, found 715.84.

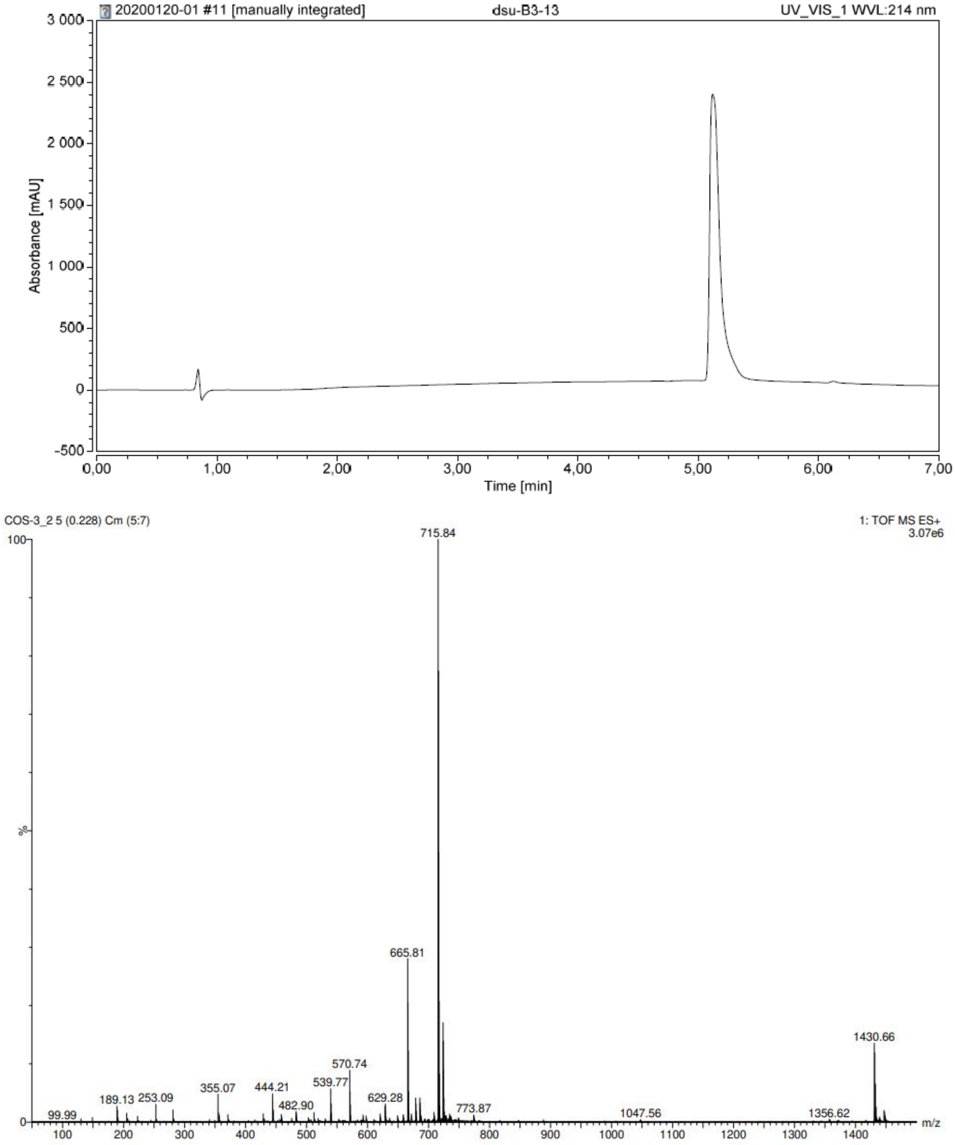

Figure S21: LC/MS analysis: UV chromatogram (top) and MS spectrum (bottom).

OxArgHyd. Yield: 57%, 207 µmol. ^{1}H NMR (400 MHz, D_2O): δ= 4.73 (s, 2H), 4.51-4.48 (m, 1H), 3.24 (t, ^{3}J = 6.8, 2H), 1.98-1.81 (m, 2H), 1.70-1.64 (m, 2H). ESI-MS m/z calcd for $[C_8H_{19}N_7O_3+H]^+$ 262.16, found 262.2.

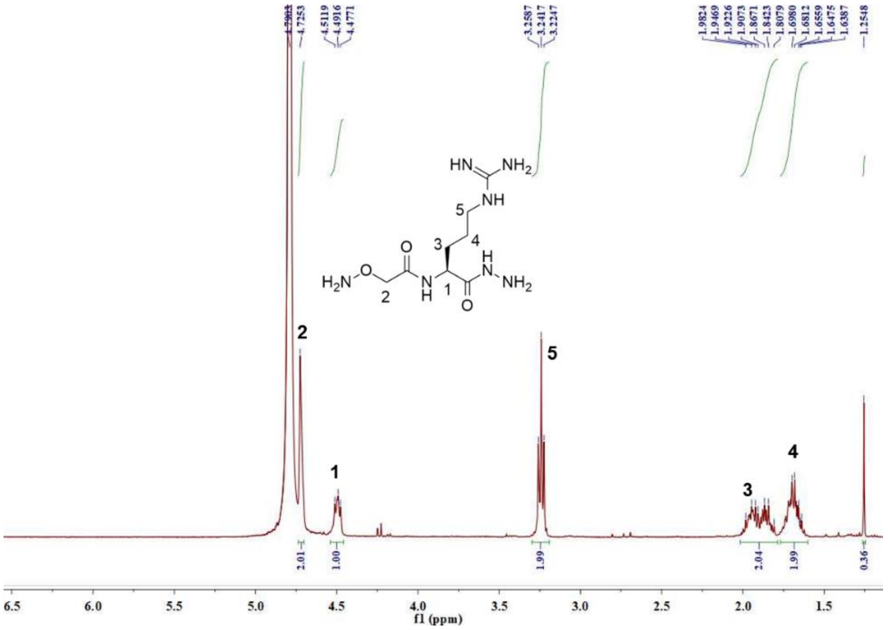

Figure S22: ^{1}H NMR (400 MHz, D_2O) spectrum.

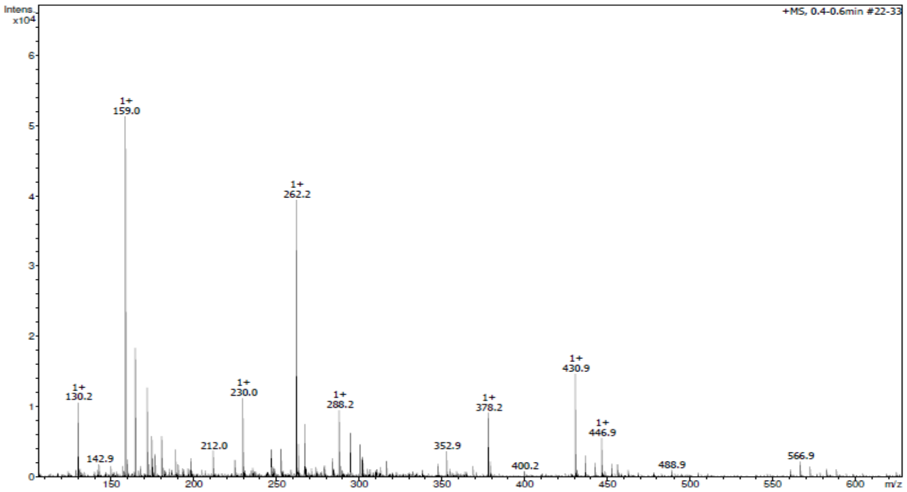

Figure S23: ESI-MS spectrum.

OxArg$_2$Hyd. Yield: 7.6%, 27.3 µmol. ^{1}H NMR (400 MHz, D_2O): δ= 4.60 (s, 2H), 4.42-4.38 (m, 2H), 3.23 (t, ^3J = 5.6, 4H), 1.93-1.79 (m, 4H), 1.75-1.62 (m, 4H). ESI-MS m/z calcd for $[C_{14}H_{31}N_{11}O_4+2H]^{2+}$ 209.63, found 209.64.

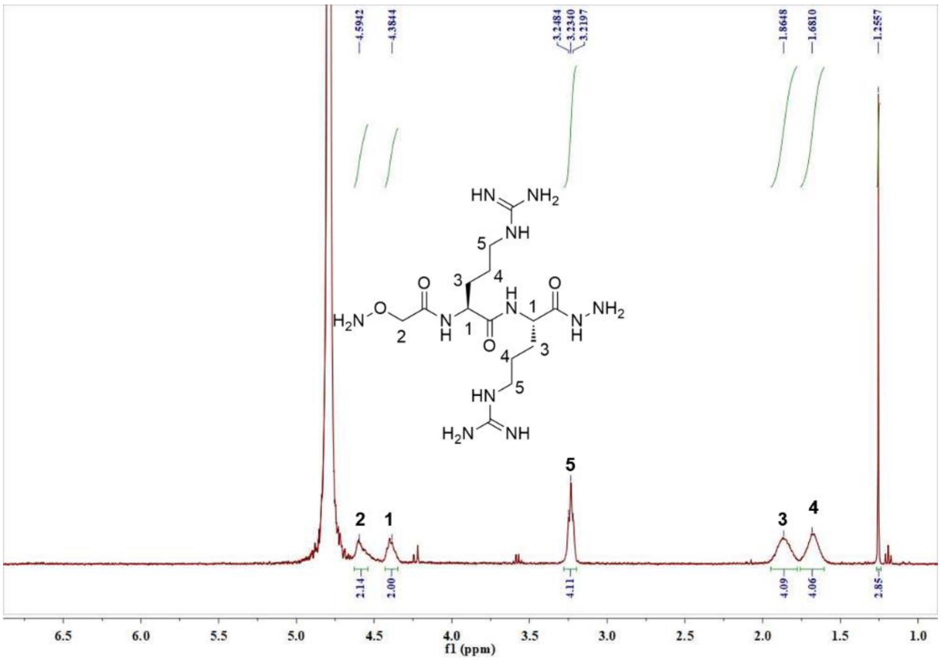

Figure S24: ^{1}H NMR (400 MHz, D_2O) spectrum.

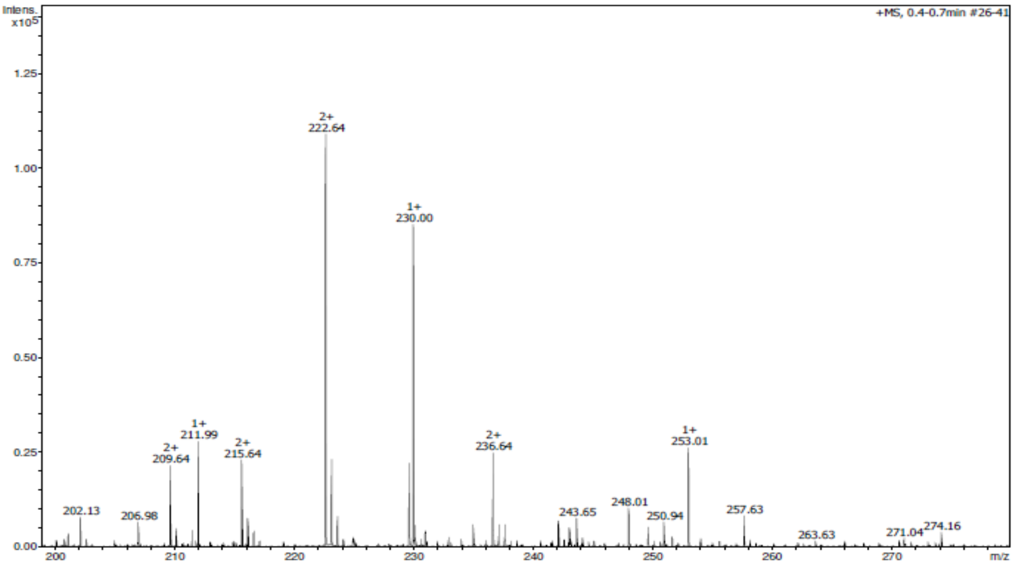

Figure S25: ESI-MS spectrum.

OxArg$_3$Hyd. Yield: 11%, 39.6 µmol. ^{1}H NMR (400 MHz, D_2O): δ= 4.34 (s, 2H), 4.07-3.98 (m, 3H), 3.22 (t, ^3J = 5.6, 6H), 1.90-1.71 (m, 6H), 1.66-1.56 (m, 6H). ESI-MS m/z calcd for $[C_{20}H_{43}N_{15}O_5+H]^+$ 574.3650, found 574.3649.

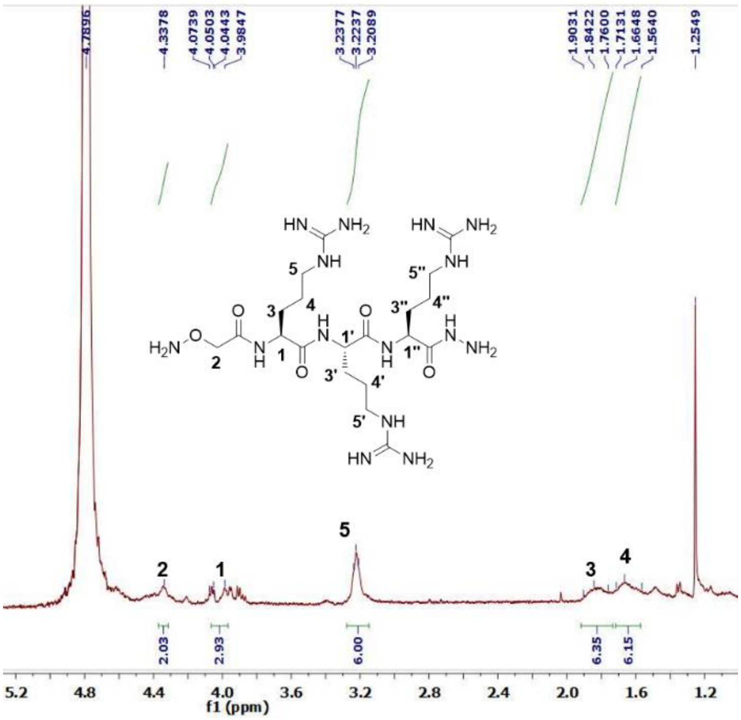

Figure S26: ^{1}H NMR (400 MHz, D_2O) spectrum.

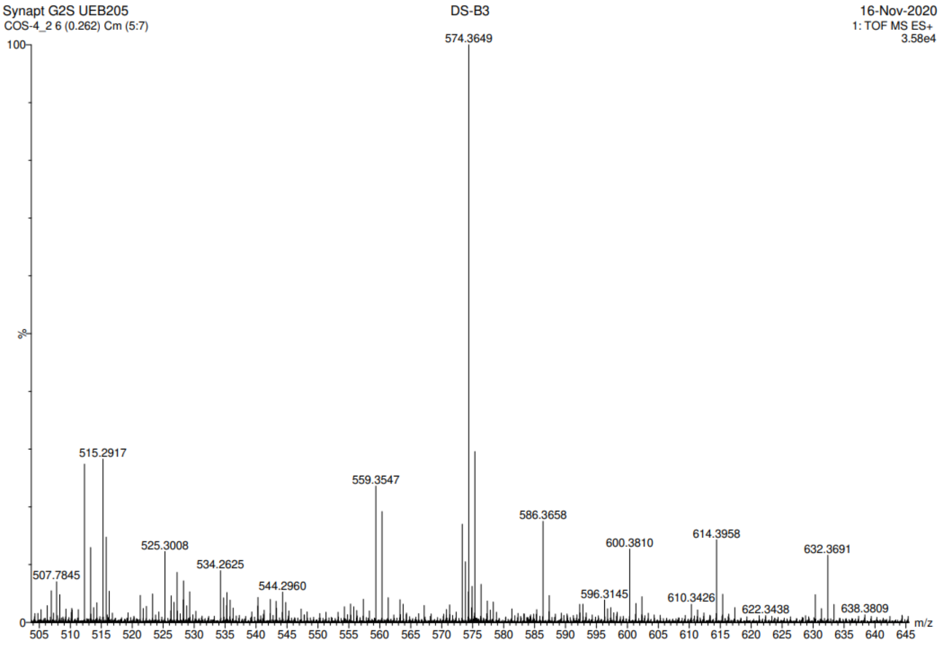

Figure S27: HR-ESI-MS spectrum.

3. Complexation of ctDNA monitored by the fluorescence displacement assay

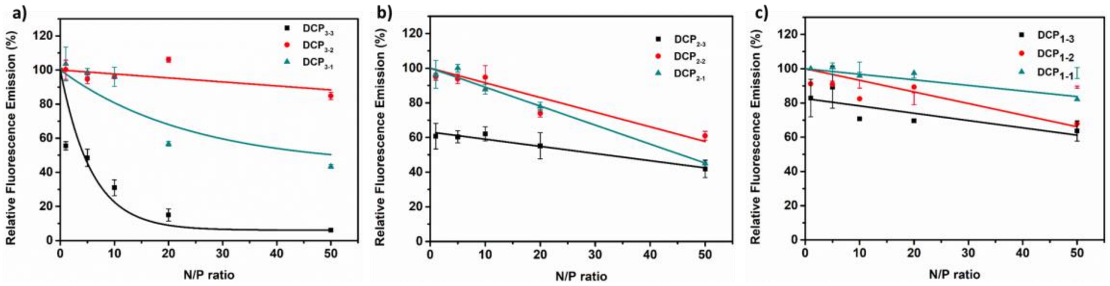

Figure S28: Relative Fluorescence Emission of the mixed solution of ctDNA and DCP_{x-y} at different N/P ratio at pH 5.5. Exponential and linear fits were used. The CE_{50} of the weak binders were determined by extrapolating the fits to a relative fluorescence emission of 50% and should therefore be taken as estimates.

4. Gel retardation assay for pDNA complexation

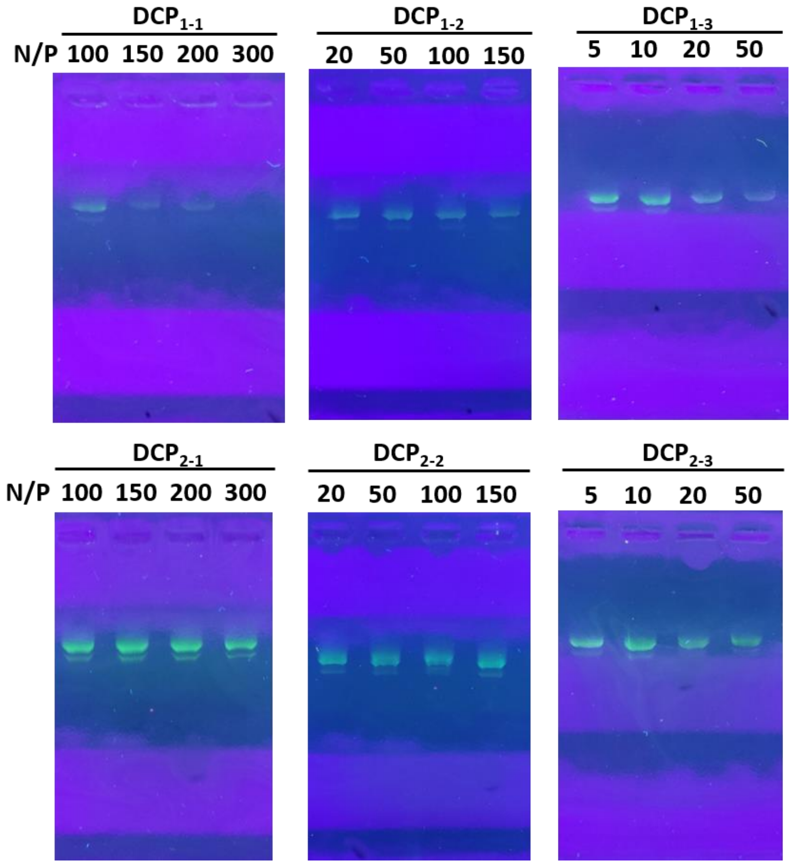

Figure S29: Gel electrophoresis of pDNA with $\mathbf{DCP_{x-y}}$ formed through *in situ* templated polymerization of monomers $\mathbf{BisAld_n}$ and $\mathbf{Ox\text{-}Arg_n\text{-}Hyd}$ at different N/P ratio.

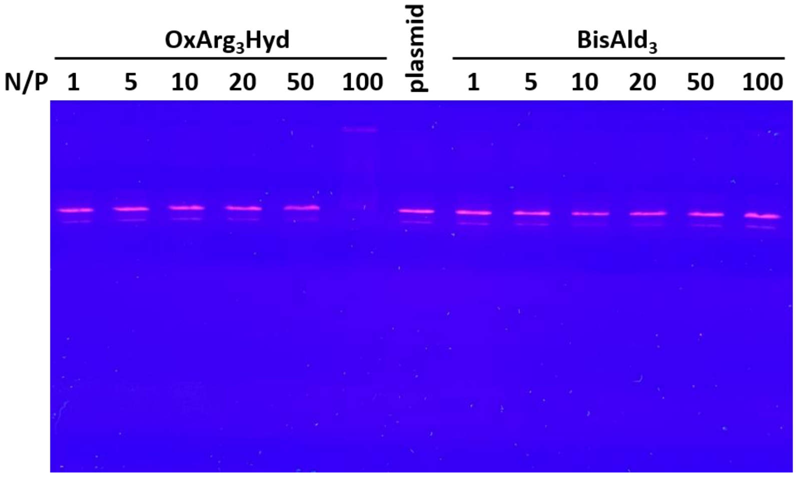

Figure S30: Gel electrophoresis of pDNA with building blocks **OxArg$_3$Hyd** and **BisAld3** at different N/P ratio at pH5.5.

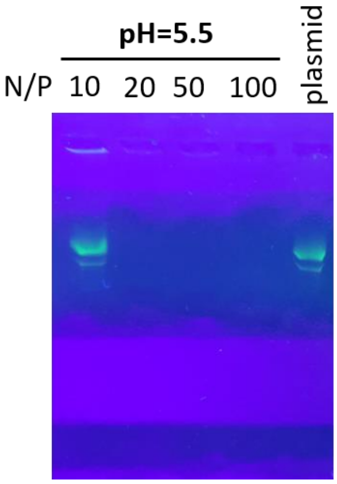

Figure S31: Gel electrophoresis of pDNA at different N/P ratio with **DCP$_{3\text{-}3}$** pre-formed at 50 mM.

5. **Gel retardation assay for siRNA complexation**

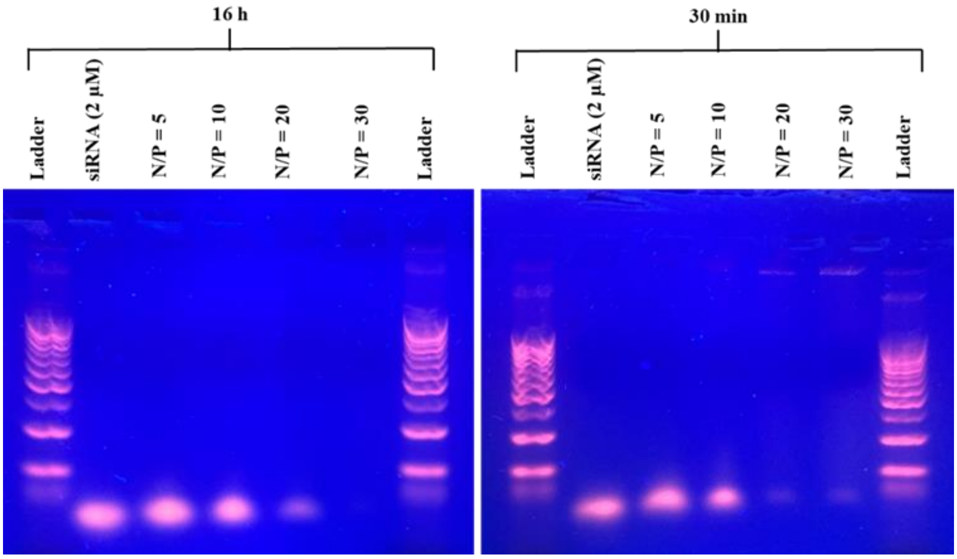

Figure S32: Gel electrophoresis of siRNA complexed with **DCP$_{3-3}$** *in situ* at different N/P ratios after 16 hours (left) and 30 minutes of incubation (right) at 25°C.

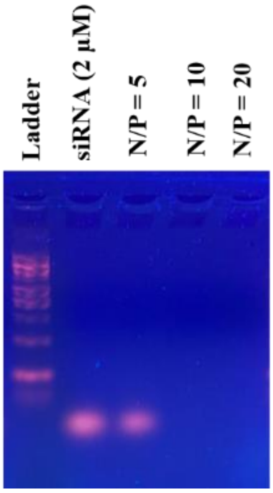

Figure S33: Gel electrophoresis of siRNA at different N/P ratio with **DCP$_{3-3}$** pre-formed at 50 mM after 16 hours of incubation at 25 °C.

6. Knock-down of luciferase activity by siRNA delivery

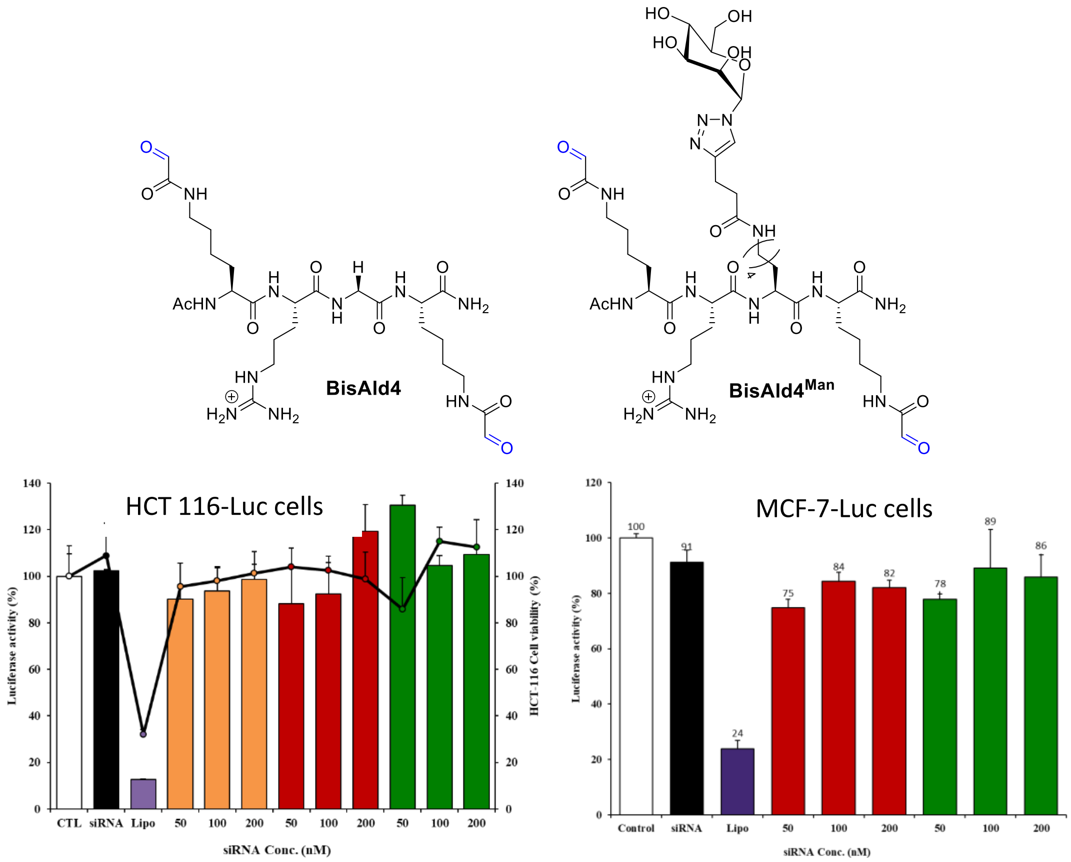

Figure S34: Chemical structures of monoarginine bisaldehyde peptides (top) and knock-down of luciferase activity by the corresponding DCPs/siLuc formed with the complementary **Ox-Arg-Hyd** peptide, on HCT116-Luc (bottom left, line represents viability) or MCF-7-Luc (bottom right) cells. The DCPs were made *in situ* from siRNA-templated polymerization as previously reported.[1] White bars: cells without any treatment; black bar: cells incubated with siLuc alone at 100 nM; colored bars: cells transfected with DCPs/siLuc at N/P = 20 with siLuc concentrations of 50, 100, and 200 nM. Blue/purple bars: lipofectamine; orange bars: **BisAld4** + **Ox-Arg-Hyd**; red bars: **BisAld4^{Man}** + **Ox-Arg-Hyd**; green bars: **BisAld4**/**BisAld4^{Man}** 8/2 + **Ox-Arg-Hyd**. The luciferase activity was adjusted depending on cell viability of each condition. The results are the average of three independent experiments and presented as mean ± SEM.

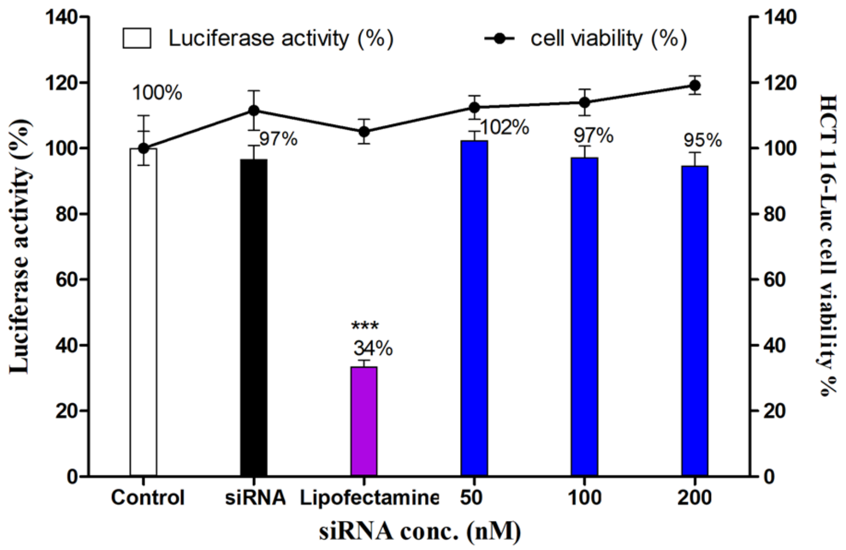

Figure S35: Knock-down of luciferase activity and cell viability using **DCP$_{1b-3}$** on HCT116-Luc cells. The DCPs were made *in situ* from siRNA-templated polymerization as previously reported.[1] White bar: cells without any treatment; black bar: cells incubated with siLuc alone at 100 nM; purple bar: lipofectamine; then blue bars: **DCP$_{1b-3}$** (N/P = 20) with siLuc concentrations of 50, 100, and 200 nM. The luciferase activity was adjusted depending on cell viability of each condition. The results are the average of three independent experiments and presented as mean ± SEM. *** statistically significant from control ($p < 0.0005$).

7. References

[1] N. Laroui, M. Coste, D. Su, L. M. A. Ali, Y. Bessin, M. Barboiu, M. Gary-Bobo, N. Bettache, S. Ulrich, *Angew. Chem. Int. Ed.* **2021**, *60*, 5783-5787.
